# P∩N Bridged Cu(I) Dimers Featuring Both TADF and Phosphorescence. From Overview towards Detailed Case Study of the Excited Singlet and Triplet States †

**DOI:** 10.3390/molecules26113415

**Published:** 2021-06-04

**Authors:** Thomas Hofbeck, Thomas A. Niehaus, Michel Fleck, Uwe Monkowius, Hartmut Yersin

**Affiliations:** 1 Institut für Physikalische Chemie, Universität Regensburg, D-93053 Regensburg, Germany; thomas@hofbeck.org; 2 Institut Lumière Matière, Univ Lyon, Université Claude Bernard Lyon 1, CNRS, F-69622 Villeurbanne, France; thomas.niehaus@univ-lyon1.fr; 3 Institut für Mineralogie und Kristallographie, Geozentrum—Universität Wien, Althanstr. 9, A-1090 Wien, Austria; michel.fleck@antonkriegergasse.at; 4 School of Education, Chemistry, Johannes Kepler University Linz, Altenbergerstr. 69, A-4040 Linz, Austria

**Keywords:** dimeric copper(I) complexes, P∩N phosphine ligands, X-ray structures, combined thermally activated delayed fluorescence (TADF) and phosphorescence, combined singlet and triplet harvesting, high emission quantum yields, tunability of photophysical properties, zero-field splitting (ZFS), spin-lattice relaxation (SLR), triplet substate decay components

## Abstract

We present an overview over eight brightly luminescent Cu(I) dimers of the type Cu_2_X_2_(P∩N)_3_ with X = Cl, Br, I and P∩N = 2-diphenylphosphino-pyridine (Ph_2_Ppy), 2-diphenylphosphino-pyrimidine (Ph_2_Ppym), 1-diphenylphosphino-isoquinoline (Ph_2_Piqn) including three new crystal structures (Cu_2_Br_2_(Ph_2_Ppy)_3_ **1-Br**, Cu_2_I_2_(Ph_2_Ppym)_3_ **2-I** and Cu_2_I_2_(Ph_2_Piqn)_3_ **3-I**). However, we mainly focus on their photo-luminescence properties. All compounds exhibit combined thermally activated delayed fluorescence (TADF) and phosphorescence at ambient temperature. Emission color, decay time and quantum yield vary over large ranges. For deeper characterization, we select Cu_2_I_2_(Ph_2_Ppy)_3_, **1-I**, showing a quantum yield of 81%. DFT and SOC-TDDFT calculations provide insight into the electronic structures of the singlet S_1_ and triplet T_1_ states. Both stem from metal+iodide-to-ligand charge transfer transitions. Evaluation of the emission decay dynamics, measured from 1.2 ≤ T ≤ 300 K, gives ∆E(S_1_-T_1_) = 380 cm^−1^ (47 meV), a transition rate of k(S_1_→S_0_) = 2.25 × 10^6^ s^−1^ (445 ns), T_1_ zero-field splittings, transition rates from the triplet substates and spin-lattice relaxation times. We also discuss the interplay of S_1_-TADF and T_1_-phosphorescence. The combined emission paths shorten the overall decay time. For OLED applications, utilization of both singlet and triplet harvesting can be highly favorable for improvement of the device performance.

## 1. Introduction

Potential applications of luminescent materials in organic light emitting diodes (OLEDs) have strongly stimulated the development of emitters that are suited for exploiting all singlet (25%) and triplet (75%) excitons [1] generated in the emission layer. Essentially, there are two different mechanisms that are already used for harvesting 100% of the excitons, namely the *triplet harvesting mechanism* [2,3,4,5,6] and the *singlet harvesting mechanism* [7,8,9,10,11,12]. For light generation based on the triplet harvesting mechanism, brightly and fast phosphorescent emitting compounds are required, such as Ir(III) or Pt(II) complexes [2,3,4,5,6,13,14,15,16,17,18,19,20,21,22,23,24,25,26,27,28]. While for singlet harvesting, the molecules have to show efficient thermally activated delayed fluorescence (TADF) at ambient temperature. Examples are found among Cu(I), Ag(I), Au(III), W(VI) and Zn(II) complexes [7,9,10,11,12,29,30,31,32,33,34,35,36,37,38,39,40,41,42,43,44,45,46,47,48,49,50,51,52,53,54,55,56,57,58,59,60,61,62,63,64,65] or specifically designed organic molecules [66,67,68,69,70,71,72,73,74,75,76,77,78,79]. For completeness, it is also referred to a very recently proposed mechanism, the *direct singlet harvesting* (DSH) mechanism [80,81]. This strategy, being based on compounds with very small energy gap of ∆E(S_1_-T_1_) ≪ k_B_T (k_B_ = Boltzmann constant), allows also for 100% exciton harvesting and additionally for drastic reduction of the emission decay time. In this report, however, we want to focus on complexes that exhibit at ambient temperature both thermally activated delayed fluorescence (TADF) and phosphorescence. Thus, they may be regarded as singlet and triplet harvesting materials. This effect of combined TADF-phosphorescence emission, suited for decreasing the overall emission decay time, has already been addressed in the literature [50,51,82,83,84,85,86,87,88,89]. In particular, Cu(I) and Ag(I) dimers, in which the metal centers are linked by P∩N ligands can show this effect [34,50,87,90,91,92,93,94,95] and very probably also the complexes reported recently [96]. To evaluate the class of materials of Cu_2_X_2_(P∩N)_3_ complexes (with X = Cl, Br, I), we study eight compounds with respect to their crystal structures and, especially, their emission properties at T = 300 K and 77 K, respectively. These data allow us to select one prominent material, Cu_2_I_2_(P∩N)_3_, **1-I** with P∩N = 2-diphenylphosphino-pyridine (Ph_2_Ppy), that shows distinct TADF and phosphorescence at ambient temperature even at high emission quantum yield of Φ_PL_ = 81% at relatively short decay time. Therefore, we investigate the emission behavior of this material as a detailed case study over the large temperature range of 1.2 ≤ T ≤ 300 K. Thus, we obtain deep insight into properties of the lowest triplet state T_1_ including its zero-field splitting (ZFS), spin-lattice relaxation (SLR) dynamics and we determine the TADF activation energy gap ∆E(S_1_-T_1_) between the lowest excited singlet state S_1_ and the T_1_ state, as well as the S_1_→S_0_ fluorescence rate. The experimental results are largely supported by SOC-TDDFT computations. Indeed, as will be shown, the combined TADF-phosphorescence decay time is distinctly shorter than the TADF-only decay. This is due to the relatively fast phosphorescence rate and the small ∆E(S_1_-T_1_) gap. Potentially, such materials showing both singlet and triplet harvesting character are attractive for applications that require short photoluminescence decay.

## 2. Results and Discussion

### 2.1. Syntheses and Structural Characterization

**Scheme 1:**
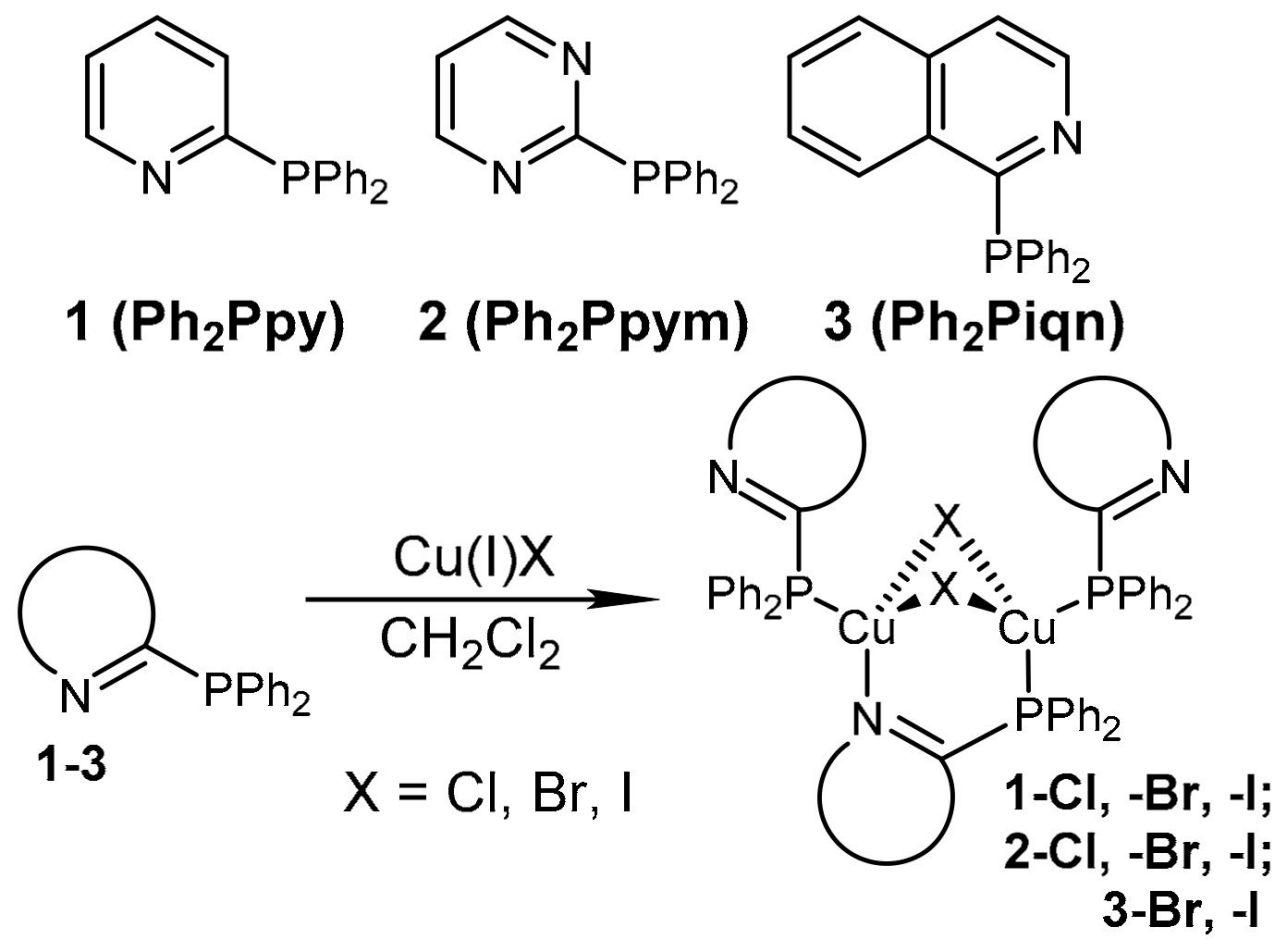
Synthesis of the di-nuclear copper complexes.

Previously, we have reported on the preparation of complexes **1-X** (X = Cl, Br, I) and **3-I** (Scheme 1) [34]. In the present work, we additionally report on the new complexes **2-X** (with X = Cl, Br, I) and **3-Br**. They were prepared analogously by reactions of the respective ligands **2** and **3** [97] with copper(I) halides in dichloromethane. For ligand **3**, the pure chloride complex could not be obtained. **1-X** and **2-X** give yellow, while **3-X** red powders. Once precipitated, the complexes are only slightly soluble in standard, weakly or non-coordinating solvents, hence, no NMR spectra could be measured. Therefore, the complexes were characterized exclusively by elemental analysis. In addition, for complexes **1-Br**, **2-I** and **3-I** the crystal structures could be determined.

**Figure 1:**
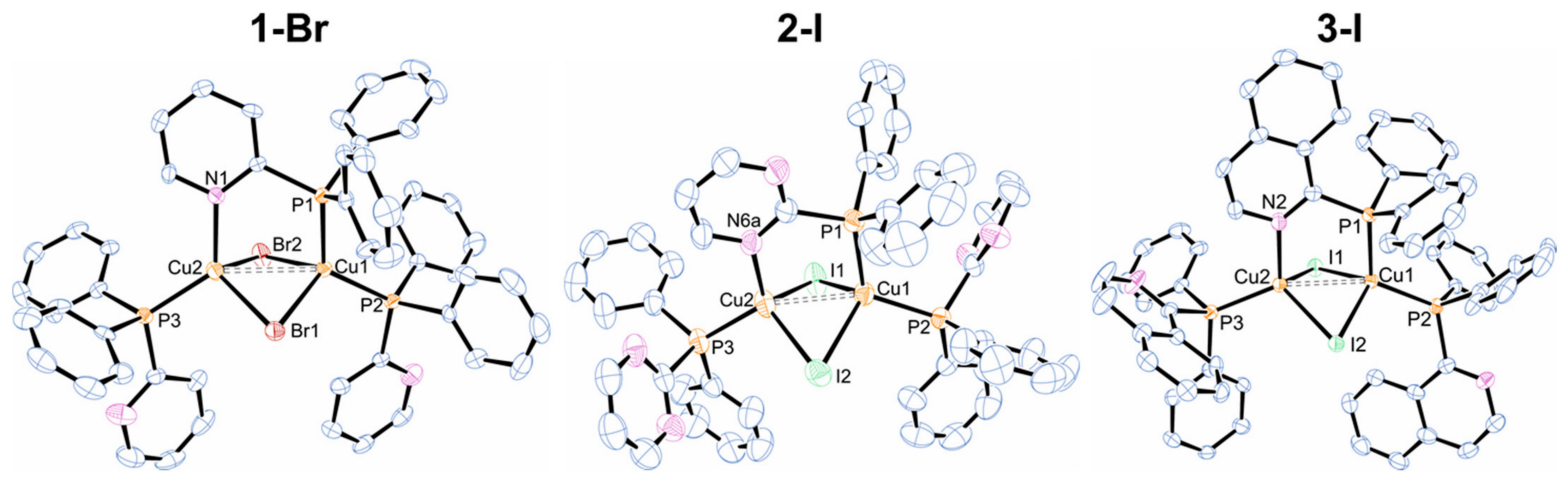
Molecular structure of Cu_2_Br_2_(Ph_2_Ppy)_3_ **1-Br**, Cu_2_I_2_(Ph_2_Ppym)_3_ **2-I** and Cu_2_I_2_(Ph_2_Piqn)_3_ **3-I** (hydrogen atoms and solvent molecules are omitted for clarity; displacement parameters are drawn at 50% probability level).

Single crystals suitable for X-ray diffraction could be obtained by slow gas-phase diffusion of diethyl ether into the filtered reaction solution of **1-Br**, **2-I** and **3-I**. To our knowledge, no bromide of this class of di-nuclear copper compounds has been structurally characterized. Therefore, the structure of **1-Br** provides the first structural data and completes the series of accessible halides. Together with the crystal structures of **1-Cl** and **1-I** reported in reference [34], it is now possible to compare all three halides of a homologous series of complexes of the type Cu_2_X_2_(P∩N)_3_. All so far structurally characterized complexes of this type have in common a butterfly-shaped Cu_2_X_2_ core surrounded by three P∩N ligands (Figure 1). Two ligands coordinate exclusively via the phosphorus atom to Cu(I), while the third ligand is bound in a bridging manner with both the nitrogen and phosphorus atoms to two Cu(I) centers.

Interestingly, not all halides **1-X** are isostructural (Table 6 below and [34]): **1-Br/Cl** crystallize in the monoclinic space group *P*2_1_/*n*, whereas **1-I** is triclinic $P\bar{1}$. Nevertheless, all structures are similar and show the expected trend for a series chloride → iodide, with only very small differences between **1-Cl** and **1-Br** (Table 1). For example, the Cu–Cu distances are almost identical for **1-Cl** (2.878(1) Å) and **1-Br** (2.883(1) Å), whereas **1-I** features a shorter Cu–Cu distances of 2.7694(5) Å. On first sight, it seems a contradictory observation that the biggest anion causes the smallest intermetallic separation. However, this fact is due the most acute angles Cu1–X–Cu2 of around 62 °C for **1-I**, whereas for **1-Cl**, we find around 73°C. The respective angle for the bromide **1-Br** lies between these values (≈69 °C). Together with a significant longer Cu–X bond distance (Cu–Cl: 2.39–2.44; Cu–Br: 2.51–2.57; Cu–I: 2.65–2.81 Å) this leads to the shortest Cu–Cu distance for **1-I**. All Cu–Cu distances are around or slightly above the sum of the van-der-Waals radii (r(Cu) = 1.40 Å [98]) indicative of only neglectable cuprophilic interactions.

Although the isoelectronic pyridyl and pyrimidyl moieties are expected to have similar steric characteristics, the structure of **2-I** (monoclinic) is not isostructural to **1-I** (triclinic) [34]. **3-I** crystallizes as the dichloromethane solvate. It should be noted that another form of complex **3-I** has been reported previously which does not differ considerably in its structural parameters (compare Table 1) [34]. For all halides and P∩N ligands summarized in Table 1, the Cu–P and Cu–N distances lie in a very narrow range of 2.24–2.29 Å and 2.08–2.14 Å, respectively.

### 2.2. Computational Investigations

**Figure 2:**
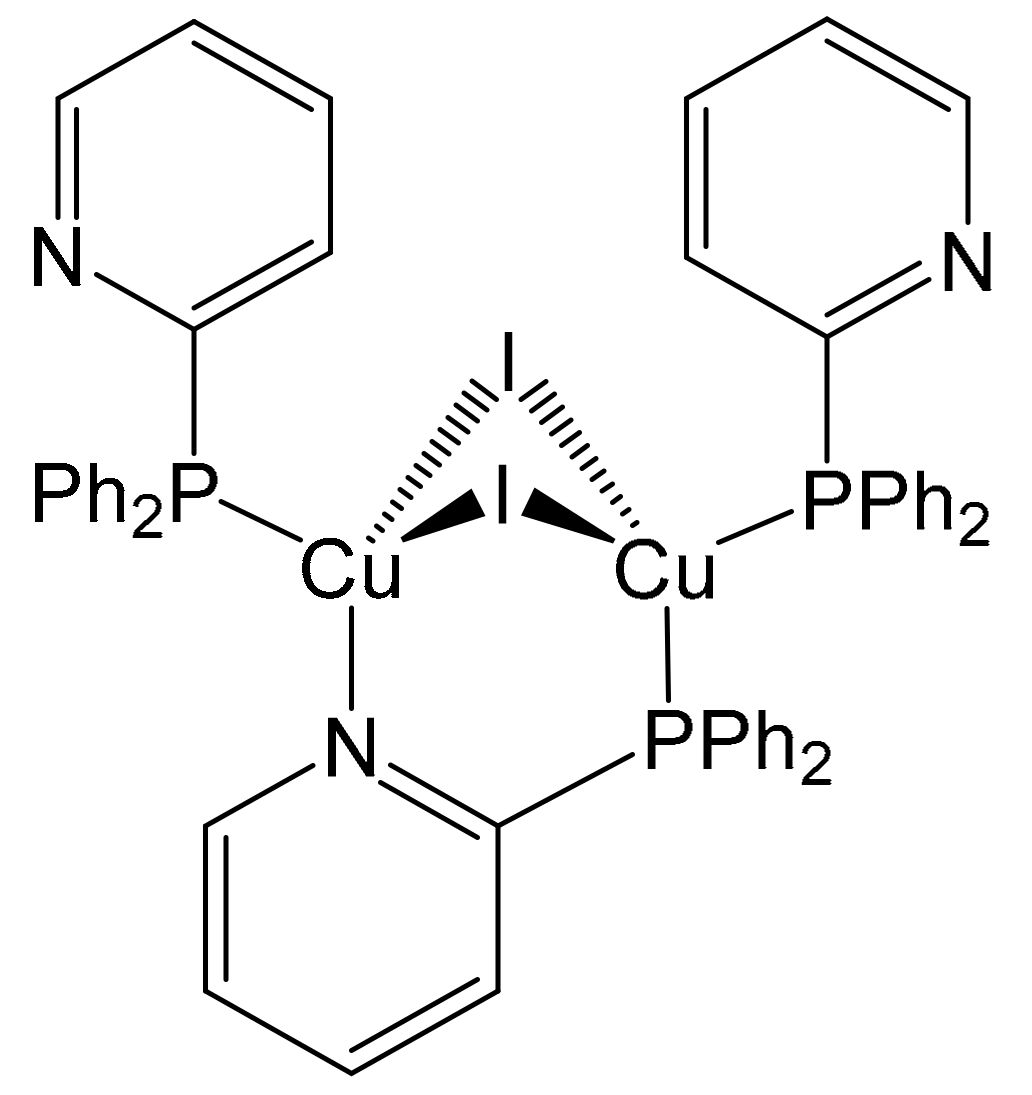
Chemical structure of Cu_2_I_2_(Ph_2_Ppy)_3_, **1-I**.

An overview of the emission data, as presented below, shows that the compound Cu_2_I_2_(Ph_2_Ppy)_3_, **1-I** (Figure 2) exhibits particularly interesting properties with respect to a distinct combination of TADF and phosphorescence and shows high emission quantum yield at short emission decay time. Accordingly, we will discuss this compound’s photophysical properties in deeper detail below. In this section, we first investigate this material by computational methods to shed light on the electronic properties.

**Figure 3:**
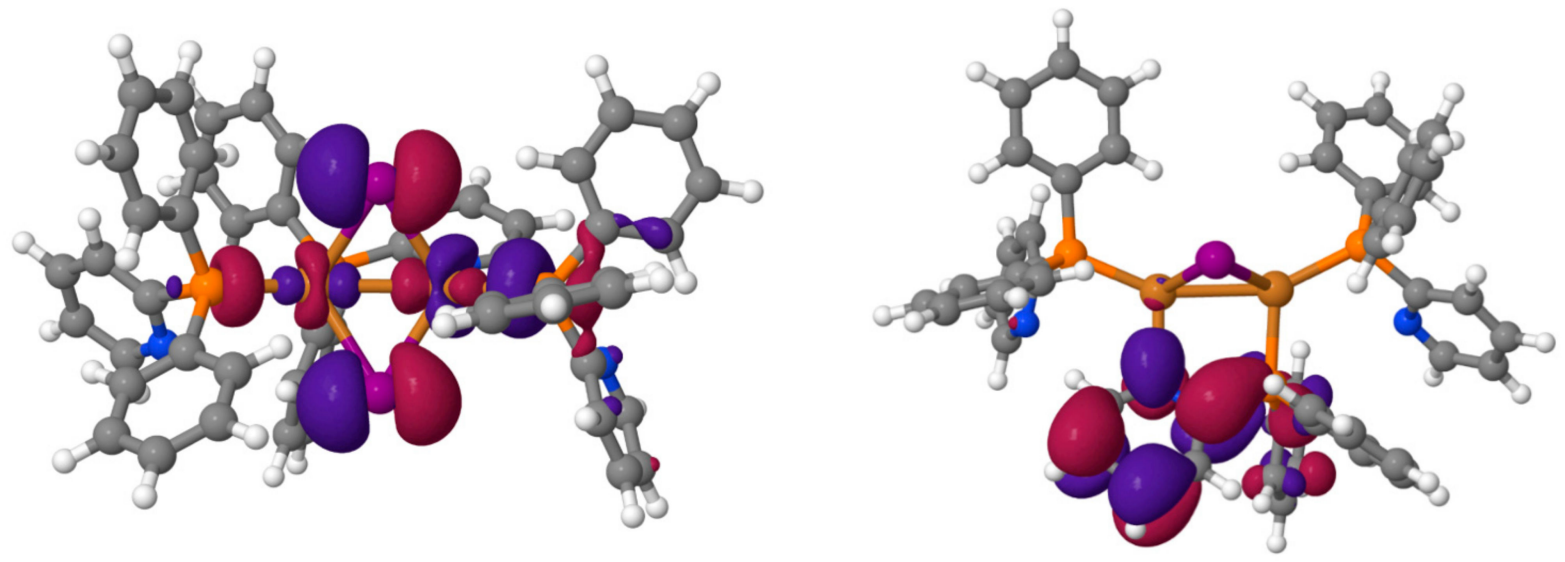
HOMO (left) and LUMO (right) of Cu_2_I_2_(Ph_2_Ppy)_3_, **1-I** at the B3LYP/DZP level for the ground state S_0_ geometry. Iso-contour values are set to 0.03 with blue/purple color representing the sign of the wave function. Color code: P (orange), Cu (brown), I (violet), N (blue), C (grey), H (white). In the right plot, the two iodine atoms are exactly on top of each other.

Initially, the compound was optimized in the singlet ground state. The calculated results reveal good agreement with the data from the X-ray structure (Table 2). Although the Cu–Cu bond length is slightly overestimated, the general structural motifs like the butterfly shape of the Cu_2_I_2_ core with an equilateral CuI_2_ triangle are nicely reproduced. Further geometry optimization was performed in the lowest triplet state using unrestricted DFT. In a next step, time-dependent DFT (TDDFT) calculations with and without self-consistent spin-orbit coupling using the ZORA Hamiltonian [99] (SOC-TDDFT [100]) were carried out for the S_0_ and T_1_ geometries with ADF2014 [101] solving for the lowest six spin-mixed excitations. Due to computational constraints, the basis set for these calculations was chosen to be DZP, a double-zeta plus polarization basis set [102]. The TDDFT calculations at the S_0_ geometry show that the S_1_ and T_1_ states correspond to a nearly pure excitation (98% and 97%) from the highest occupied molecular orbital (HOMO) to the lowest unoccupied molecular orbital (LUMO) as similarly reported in [34]. As depicted in Figure 3, the HOMO is localized on the Cu_2_I_2_ core with significant contributions of the iodides, while the LUMO is localized on the bridging organic ligand. This leads to a classification of the HOMO-LUMO transition as being of (I+M)LCT character, abbreviated shortly as metal-to-ligand charge transfer (MLCT) transition.

Taking spin-orbit coupling (SOC) into account, also the energetic separations between the three triplet substates, the zero field splittings (ZFSs) and the radiative lifetimes of these states can be assessed (Table 3). At the S_0_ geometry, a moderate agreement with the experimental data is observable. The calculated vertical excitation energies are somewhat underestimated and the small computed singlet-triplet splitting, although predicting **1-I** as a TADF material, underestimate the experimental ∆E(S_1_-T_1_) gap. Clearly, several important aspects are not taken into account at this level of theory. First, the inclusion of the difference in zero-point energies of ground and excited state may alter transition energies by 0.1–0.2 eV, usually leading to red shifted emission energies. Second, one generally expects excited state geometry relaxation to occur [103,104,105]. To investigate its impact, simulations were also carried out at the optimized T_1_ state geometry. As Table 3 shows, this leads to a strongly red shifted emission, probably being significantly influenced by the strong shortening of the Cu–Cu bond length (Table 2). Experimentally, a strong dependence of the emission wavelength on the molecular environment was observed. For example, related Cu_2_I_2_(P∩N)_3_ [(P∩N) = (2-diphenylphosphino)-4-alkyl-pyridine] complexes, as presented in [34], show strong red shifts going from the powder material to PMMA (poly(methyl methacrylate)) matrices and, finally, to solvents (also compare [37]). In the latter environments, large scale atomic rearrangements can easily occur. We find that the computed bond length reduction at the T_1_ optimized geometry is accompanied by a 30 °C rotation of the P∩N ligand around the Cu1–P2 axis (Table 2). While the former geometry change could also be realized in a polycrystalline environment, the latter one would likely be sterically hindered. Another influence is the effect of the dielectric environment, which probably is important due to the charge transfer character of the emission. Since a realistic modeling of the environment of the complex within the powder material is challenging, we performed SOC-TDDFT calculations with the COSMO continuum [106] solvent model (parameters: dielectric constant of dichloromethane (DCM) ε = 8.9, radius of solvent molecules r(sol) = 2.94 Å) for the S_0_ geometry to obtain information about the general trends. Estimates of the radiative lifetimes are based on transition energies and transition dipole matrix elements as outlined in [107]. Corrections due to the refractive index of the molecular environment (n(DCM) = 1.42) were accounted for by the empty spherical cavity model [108]. Table 3 reveals a strong blue shift of ≈0.4 eV (≈3000 cm^−1^) compared to a gas phase environment. The observed blue shift can probably be explained by the considerable dipole moment change by the charge transfer from the Cu_2_I_2_ core to the bridging ligand.

The calculated data, presented in Table 3, show considerably different energies, depending on the model and the state geometry chosen [109]. For comparison to emission data, we focus on the T_1_ state geometry. Although the calculated transition energies are underestimated (in gas phase environment), it is usually accepted that energy differences display the experimental situation more realistically. The corresponding emission data are presented below in Section 2.4 and in Figure 8. It is seen that the energy gap ∆E(S_1_-T_1_) is very well reproduced in the T_1_ state geometry, while the ZFS value ∆E(III–II,I) is overestimated. This might be related to the model restricted to a too small number of spin-state-mixing of higher lying states. However, the very small splitting of ∆E(II–I) or the almost degenerate situation for the two low-lying triplet substates I and II is well reproduced by the SOC-TDDFT model (Table 3 and Figure 8, below).

### 2.3. Luminescence Properties of Cu_2_X_2_(P∩N)_3_ Complexes with X = Cl, Br, I. An Overview

In this section, we will discuss the emission behavior of eight Cu_2_X_2_(P∩N)_3_ complexes studied at 300 K and 77 K and give an overview over the large scale of varying emission properties.

The complexes studied are not soluble in common, non-coordinating organic solvents. Therefore, all measurements were performed with powders. Usually, investigations of solid-state samples are not convenient for studies of detailed emission properties, due to the influence of processes like triplet-triplet annihilation or energy transfer. However, for Cu(I) complexes, usually a self-trapping mechanism takes place that leads to quasi-isolated molecules embedded in the neat material without any significant excited state resonance interaction with the environment [37,110,111].

**Figure 4:**
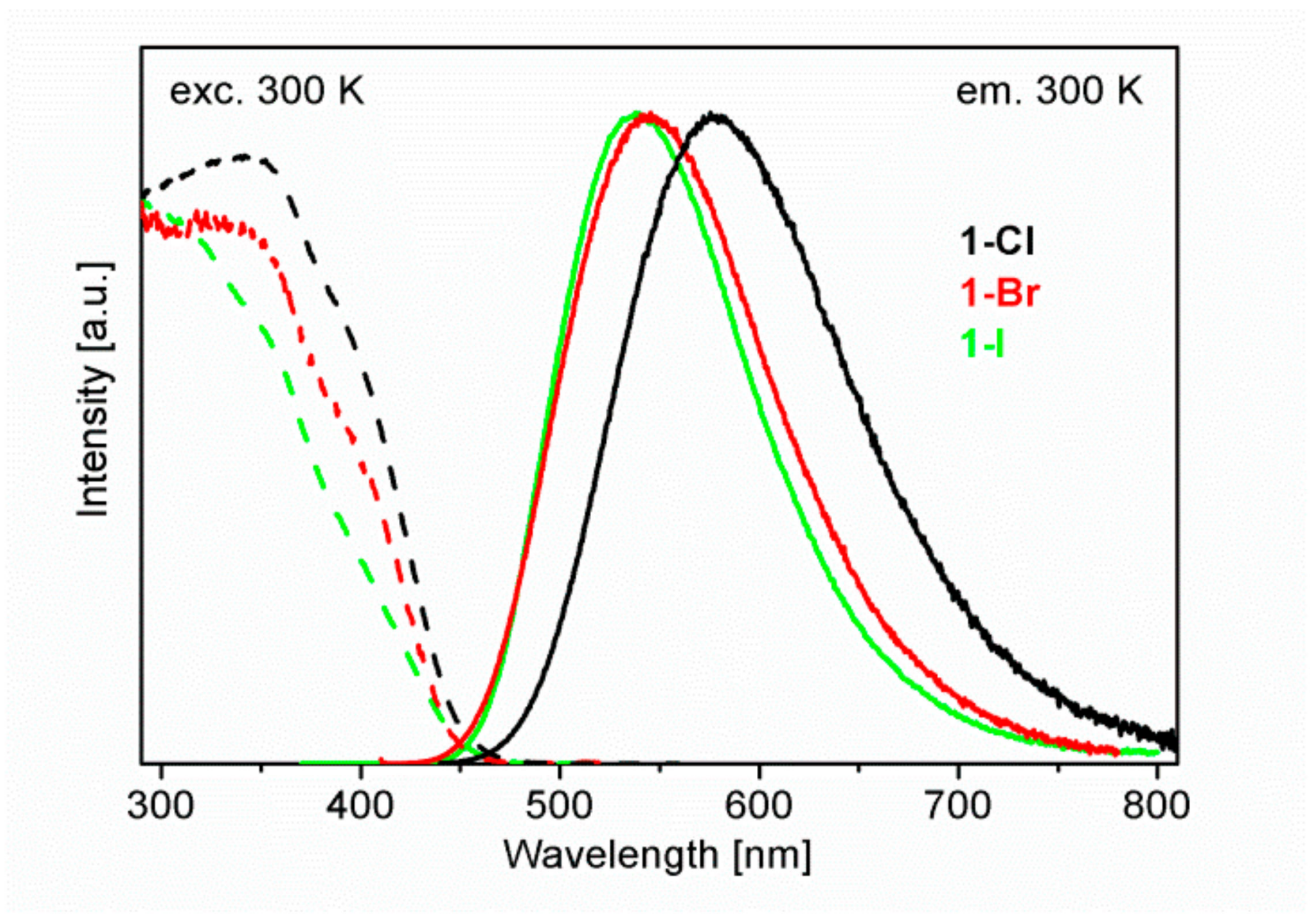
Emission and excitation spectra of Cu_2_Cl_2_(Ph_2_Ppy)_3_, **1-Cl**, Cu_2_Cl_2_(Ph_2_Ppy)_3_, **1-Br** and Cu_2_I_2_(Ph_2_Ppym)_3_, **1-I** (powder, 300 K, λ_exc_ = 350 nm).

As summarized in Table 4, all studied di-nuclear Cu_2_X_2_(P∩N)_3_ complexes show relatively intense photoluminescence under UV excitation with broad emission bands in the green to red spectral range assigned to (iodide+metal)-to-ligand charge transfer, (I+M)LCT transitions, as predicted by DFT calculations presented in the previous section. The HOMO resides on the Cu(I)-halide core, while the LUMO is largely localized on the bridging (P∩N) ligand (Figure 3). Thus, modification of the ligand, in particular, of an extension of the ligand’s aromatic system leads to a red shift of the MLCT transition [34]. There is a clear trend of the emission maxima depending on the halides. In each series, the emission maxima are blue shifted from Cl to Br to I complexes. For example, Figure 4 shows the emission spectra of the **1-X** series. For ambient temperature, the peak maxima are blue shifted from **1-Cl** to **1-I** by about 1200 cm^−1^. The flank of the excitation spectra in the region above about 400 nm shows a similar trend. This observation is rationalized by a reduction of the ligand field strength of the halides in the series Cl^−^ > Br^−^ > I^−^ (being contrary to the trend of electronegativity) and thus, by a larger HOMO-LUMO energy gap [34,112]. The photoluminescence quantum yields at T = 300 K lie between Φ_PL_ = 9% and 81%. At ambient temperature, the emission decay times are found in the lower µs time regime between 1.2 to 8.8 µs. However, with respect to photophysical interpretations, it is usually better to compare radiative decay times τ^r^ = τ/Φ_PL_. In the series of compounds given in Table 4, they lie between 4.7 × 10^4^ s^−1^ (21 µs) and 1.25 × 10^5^ s^−1^ (8 µs).

Upon cooling to 77 K, the emission quantum yields increase remarkably, for example, for compound **2-I** by a factor of more than five. Such a behavior is not unusual, since non-radiative deactivation processes frequently become less important with temperature reduction [113]. Moreover, the emission bands of all complexes are red shifted. Most affected is the **1-X** series with shifts of about 440 cm^−1^ (54 meV, **1-Cl** and **1-I**) and about 710 cm^−1^ (88 meV, **1-Br**), while for the compounds **2-X** and **3-X**, values below around 300 cm^−1^ (37 meV) are observed. These energy separations frequently display approximately the energy gap ∆E(S_1_-T_1_) that is responsible for thermal activation of the TADF emission. Interestingly, the value of 440 cm^−1^ (54 meV) found for Cu_2_I_2_(Ph_2_Ppy)_3_, **1-I** corresponds well to the calculated one of 411 cm^−1^ (51 meV, Table 3). Concomitantly, the radiative rates of the emission decrease strongly (increasing decay times) with cooling, for example, by a factor of about 16 for compound **2-Cl**. These effects, red shift and radiative rate decrease, occurring upon cooling, are consequences of freezing out the additional emission decay channel via the energetically higher lying S_1_ state. In other words, TADF emission is largely frozen out. Thus, at sufficiently low temperature, mostly at T = 77 K but sometimes only below T = 50 K [29], all compounds exhibit only phosphorescence.

**Figure 5:**
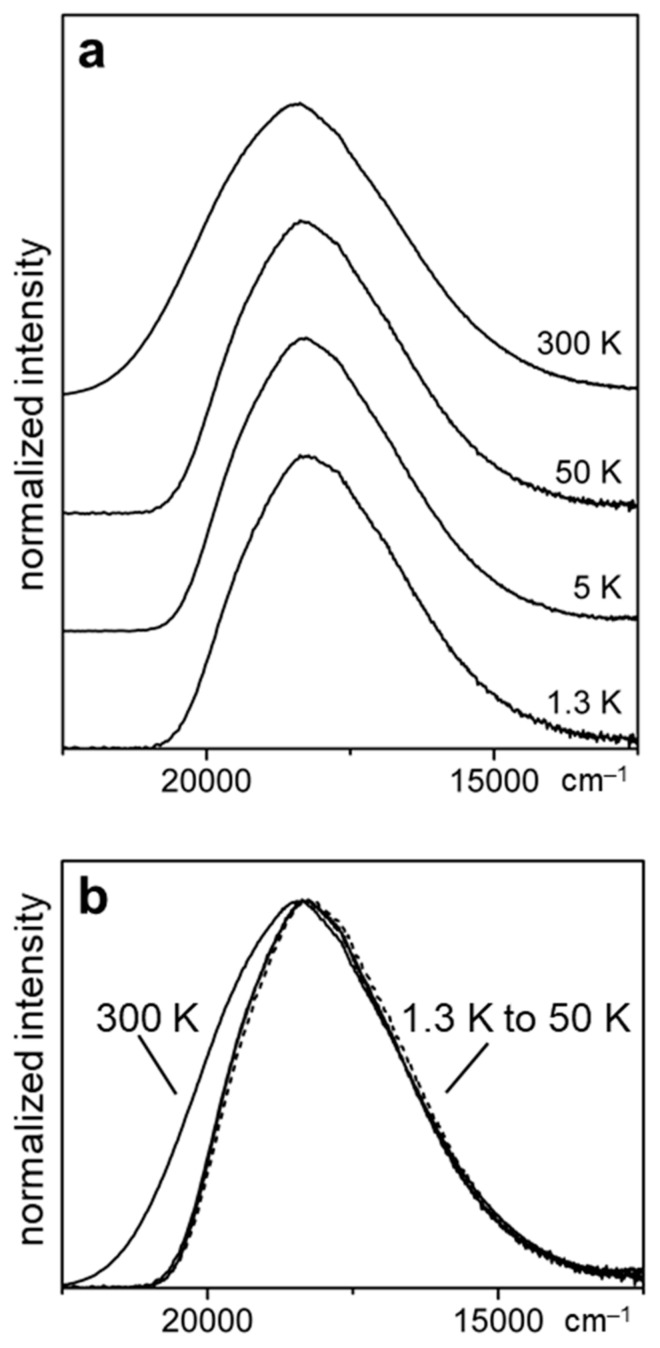
Emission spectra of Cu_2_I_2_(Ph_2_Ppy)_3_, **1-I** at different temperatures (powder, λ_exc_ = 355 nm). The spectra for different temperatures shown in (**a**) are super-imposed in (**b**) to visualize the intensity growing in at the blue side flank with temperature increase.

The spectral changes of the emission bands with temperature variation are shown in Figure 5a for compound **1-I**. At low temperature (1.3 K to almost 77 K), only T_1_→S_0_ phosphorescence is occurring, while with further temperature increase to T = 300 K, the emission band center is blue shifted, mainly observable by a blue-side flank growing in above around T = 70 K (Figure 5b). The corresponding additional band is assigned to the thermally activated fluorescence from the higher lying S_1_ state. Apparently, already the spectral changes displayed in Figure 5 indicate that at ambient temperature, the emission consists of overlapping TADF and phosphorescence. The appearance of a phosphorescence contribution even at ambient temperature is additionally based on the relatively fast radiative rate of k^r^(T_1_→S_0_, 77 K). Such a behavior of combined TADF and phosphorescence is not very frequently observed [48], but see refs. [50,51,83,86,114]. Mostly, however, the TADF channel dominates strongly [48]. For a more detailed discussion see Section 2.4.

Of the series of compounds studied (Table 4), especially, the iodide containing complexes **1-I**, **2-I** and **3-I** show fast radiative phosphorescence rates lying above k^r^(T_1_→S_0_) = 2 × 10^4^ s^−1^ (50 µs). This may be rationalized by two factors. First, significant SOC is induced by admixtures of higher lying singlet states S_n_ to the T_1_ state. Secondly, the large contributions of 5p-orbitals of iodide to the higher lying occupied orbitals (Figure 3) with the high SOC constant of iodide of ξ(I) = 5069 cm^−1^ [115] may also play a role in speeding up the T_1_→S_0_ transition rate [116]. This latter effect is supported by the distinctly lower T_1_→S_0_ rate found for the chloride compounds with ξ(Cl) = 587 cm^−1^ [115] (Table 4).

A deeper discussion of the data summarized in Table 4 will not give more detailed information, in particular, not the spectral features available. This is a consequence of the very broad emission bands of MLCT character with halfwidths of ≈3500 cm^−1^ (440 meV) even at T = 1.3 K. On the other hand, investigation of the emission decay behavior with temperature variation will lead to a detailed characterization of the compounds’ electronic structure, especially, of the lowest excited states, as will be shown in the next section.

### 2.4. Detailed Case Study of Cu_2_I_2_(Ph_2_Ppy)_3_, 1-I. the Lowest Excited Triplet and Singlet States

For a deeper case study, we selected Cu_2_I_2_(Ph_2_Ppy)_3_, **1-I** (powder) due to four reasons. First, this material exhibits the highest emission quantum yield of Φ_PL_ = 81% of all compounds summarized in Table 4. Second, **1-I** emits with one of the fastest radiative phosphorescence rate of k^r^(77 K) = 2.88 × 10^4^ s^−1^ (τ^r^(77 K) = 35 µs). Third, the emission decay time is mono-exponential over the whole temperature range (apart from very low temperature) and fourth, the emission quantum yield changes only slightly with temperature. These two latter properties are required for a detailed characterization based on the τ(T) fitting procedure as discussed below. With respect to these properties, **1-I** represents a remarkable material and is expected to show clearly the interplay of phosphorescence and TADF. Moreover, according to the fast rate, efficient SOC experienced by the T_1_ state should lead to a well observable zero-field splitting (ZFS) of T_1_ into substates I, II and III, as already predicted by the SOC-TDDFT calculations (Section 2.2). This should lead to specific relaxation properties within the manifold of the substates, in particular, such as effects of spin-lattice relaxation (SLR) [117].

**Figure 6:**
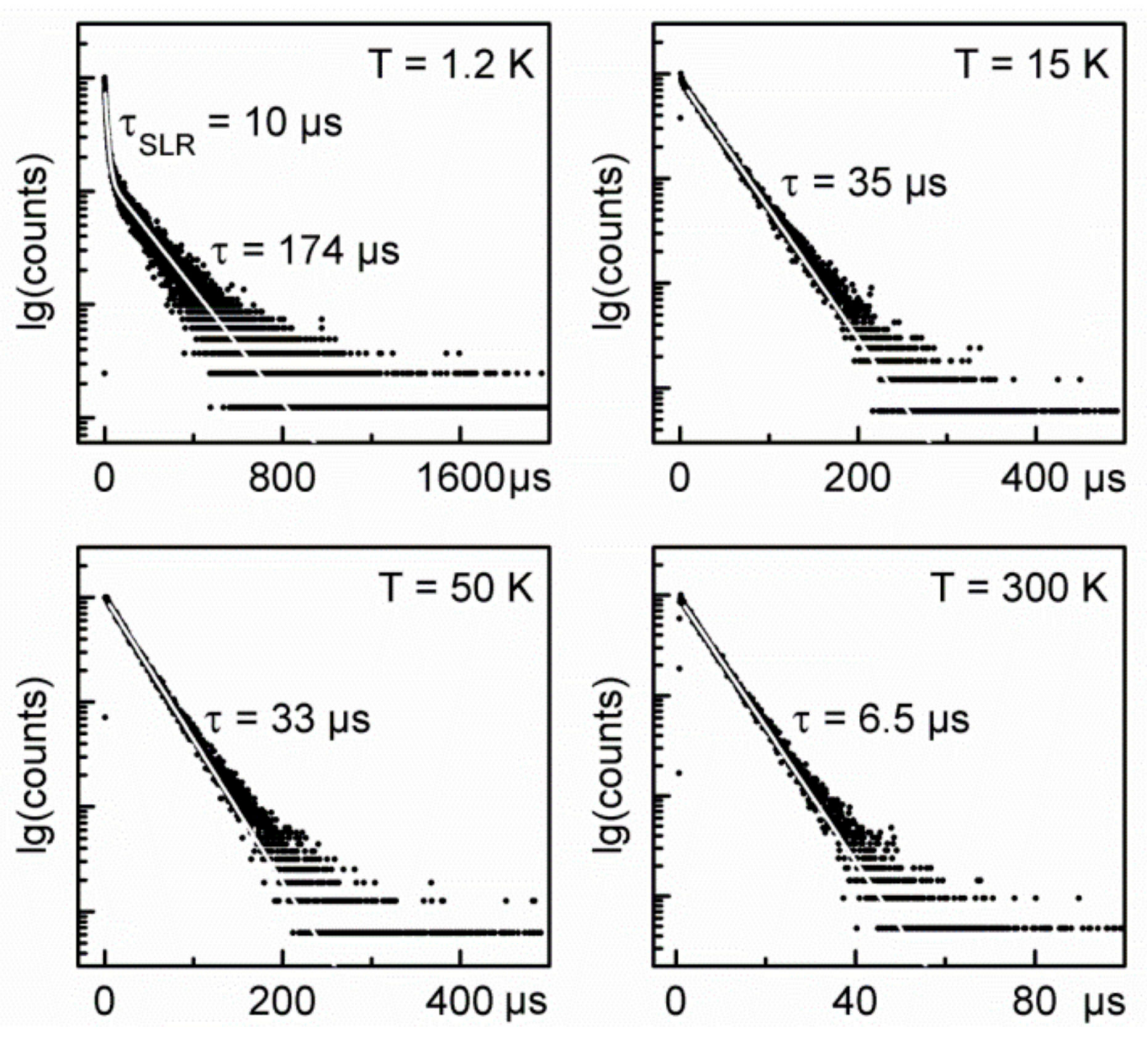
Emission decay behavior of Cu_2_I_2_(Ph_2_Ppy)_3_, **1-I** at different temperatures (powder, λ_exc_ = 355 nm). At 1.2 K, a bi-exponentially decay can be observed showing a short component that is determined by spin-lattice relaxation (SLR) processes between the triplet substates. The long component represents the decay of the involved thermalized states. At higher temperatures, the decay is mono-exponential. Note the different scales.

For the detailed characterization of the compound’s electronic structure and the related decay rates, we study the emission decay behavior of **1-I** over the large temperature range of 1.2 ≤ T ≤ 300 K. Figure 6 displays selected emission decay curves measured at different temperatures. At T = 1.2 K, the emission decays bi-exponentially with two clearly different components of 10 µs and 174 µs, respectively. Such a behavior can be well rationalized, if the lowest triplet state exhibits distinct ZFS. Thus, the short component refers to spin-lattice-relaxation (SLR) processes within the triplet substate manifold [117], while the longer component is ascribed to the thermalized emission that is established after around 60 µs at T = 1.2 K (estimated from the 1.2 K decay curve reproduced in Figure 6). It is known that thermalization according to SLR processes is strongly temperature dependent and becomes faster with temperature increase [117]. Indeed, the observed short decay component decreases from 10 µs at T = 1.2 K to 1.7 µs at T = 10 K and is faster than detectable (with our equipment) at T = 15 K (Figure 6). This means that SLR processes are getting much faster than the thermalized emission decay time. Therefore, shortly after the excitation pulse, the population numbers of the excited states behave according to a Boltzmann distribution. We will come back to SLR properties below in this section.

**Figure 7:**
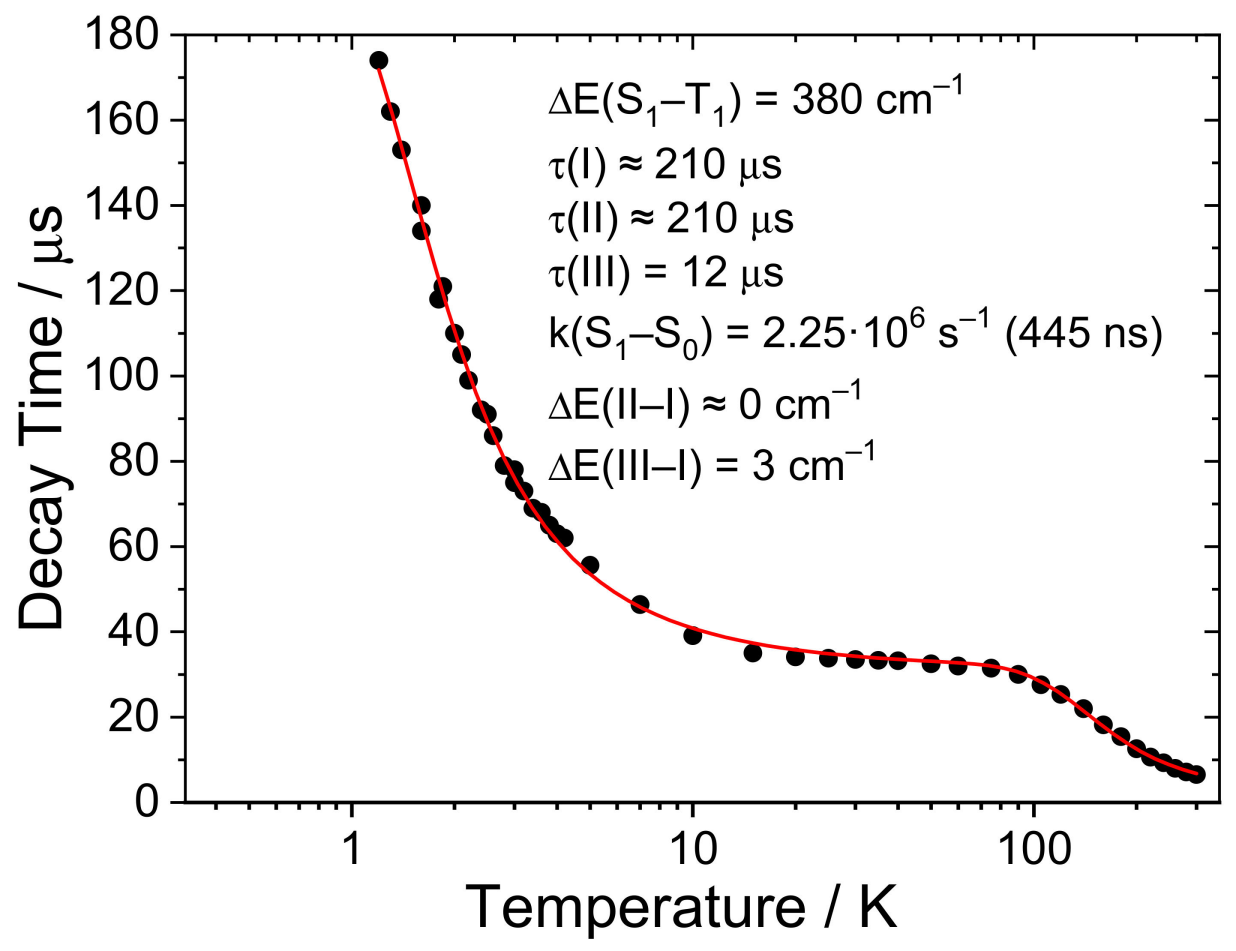
Temperature dependence of the emission decay time of the thermally equilibrated excited states of Cu_2_I_2_(Ph_2_Ppy)_3_ **1-I**. The calculated fit is based on Equation (1). As inset, we summarize the fit parameters.

The decay time of the long (thermalized) component is also drastically shortened with temperature increase (Figure 7). Due to the photophysical mechanisms involved, the temperature dependence can be classified into three ranges: (i) Up to T ≈ 20 K, the decay behavior is determined by the zero-field split T_1_ substates. At T = 1.2 K, we find a long component of τ = 174 µs. This emission dominantly stems from the thermally equilibrated two lower triplet substates I and II. By temperature increase the decay is strongly shortened (by a factor of more than five) when reaching a plateau near 20 K. The plateau decay time amounts to about 32 µs. This decrease is resulting from population of the higher lying triplet substate III. Because this transition III(T_1_)→S_0_ exhibits a much faster rate than the rates from the two lower lying substates I and II, an additional decay path is opened. (ii) Within the temperature range of the plateau, between about 20 K and about 70 K, the triplet substates I, II and III are thermally equilibrated and emit as T_1_→S_0_ phosphorescence with an average decay time of the three substates of 32 µs (see also Equation (4), below). (iii) With further temperature increase from T ≈ 70 K to 300 K, the decay time decreases to τ(300 K) = 6.5 µs. In this temperature range, the photoluminescence quantum yield changes only slightly (Table 4). Hence, the decay time decrease can (largely) be ascribed to a rate increase by a factor of more than four from k^r^(77 K) = 2.88 × 10^4^ s^−1^ to k^r^(300 K) = 12.5 × 10^4^ s^−1^ (Table 4). This increase is induced by opening the TADF decay path via the S_1_→S_0_ transition in addition to the still remaining phosphorescence decay path (see also below).

For the mono-exponential ranges of the decay curves (Figure 6), it can be concluded that the emitting states are in fast thermal equilibration with respect to the individual decay times of the states involved. This is not only valid for the SLR processes but also for down- and up-inter-system crossing (ISC) processes between the T_1_ and the S_1_ states. Down-ISC will probably occur within around 10 ps or even shorter [103,104,105,118,119,120,121], while the up-ISC time (also named RISC time) is strongly temperature dependent [122] and may be estimated very roughly to around 30 ns at 80 K and to about 0.2 ns at ambient temperature. For completeness, it is mentioned that very fast ISC processes are probably based on direct SOC of higher lying singlet states to T_1_ substates as is displayed in a high allowedness of the T_1_→S_0_ transition. This is in contrast to the situation of molecules with weak SOC with respect to the lowest triplet state, as it seems to be valid for most organic molecules. For these, spin-vibronic processes will probably dominate the ISC rate [123].

Thus, with respect to the emission decay times of many µs, fast thermalization is well realized for the Cu(I) compounds discussed here. Accordingly, it is justified to describe the temperature dependence of the emission decay time τ(T), as shown in Figure 7, by a modified Boltzmann distribution of the four thermally equilibrated excited states, the three T_1_ substates I, II and III and the S_1_ state [19,50,87,124].

(1) $$\tau \left(T\right)=\frac{1+\exp\left(-\frac{\Delta E\left(\mathrm{II}-I\right)}{k_{B}T}\right)+\exp\left(-\frac{\Delta E\left(\mathrm{III}-I\right)}{k_{B}T}\right)+\exp\left(-\frac{\Delta E\left(S_{1}-I\right)}{k_{B}T}\right)}{k\left(I\right)+k\left(\mathrm{II}\right)\exp\left(-\frac{\Delta E\left(\mathrm{II}-I\right)}{k_{B}T}\right)+k\left(\mathrm{III}\right)\exp\left(-\frac{\Delta E\left(\mathrm{III}-I\right)}{k_{B}T}\right)+k\left(S_{1}\right)\exp\left(-\frac{\Delta E\left(S_{1}-I\right)}{k_{B}T}\right)}$$

Herein, ∆E(II–I) and ∆E(III–I) are the ZFS values and ∆E(S_1_–I) is the energy gap between the S_1_ state and the T_1_ state (for ∆E(S_1_–T_1_) >> ∆E(III–I)). k(I), k(II), k(III) and k(S_1_) are the transition rates of the respective states to the electronic ground state S_0_. k_B_ is the Boltzmann constant.

**Figure 8:**
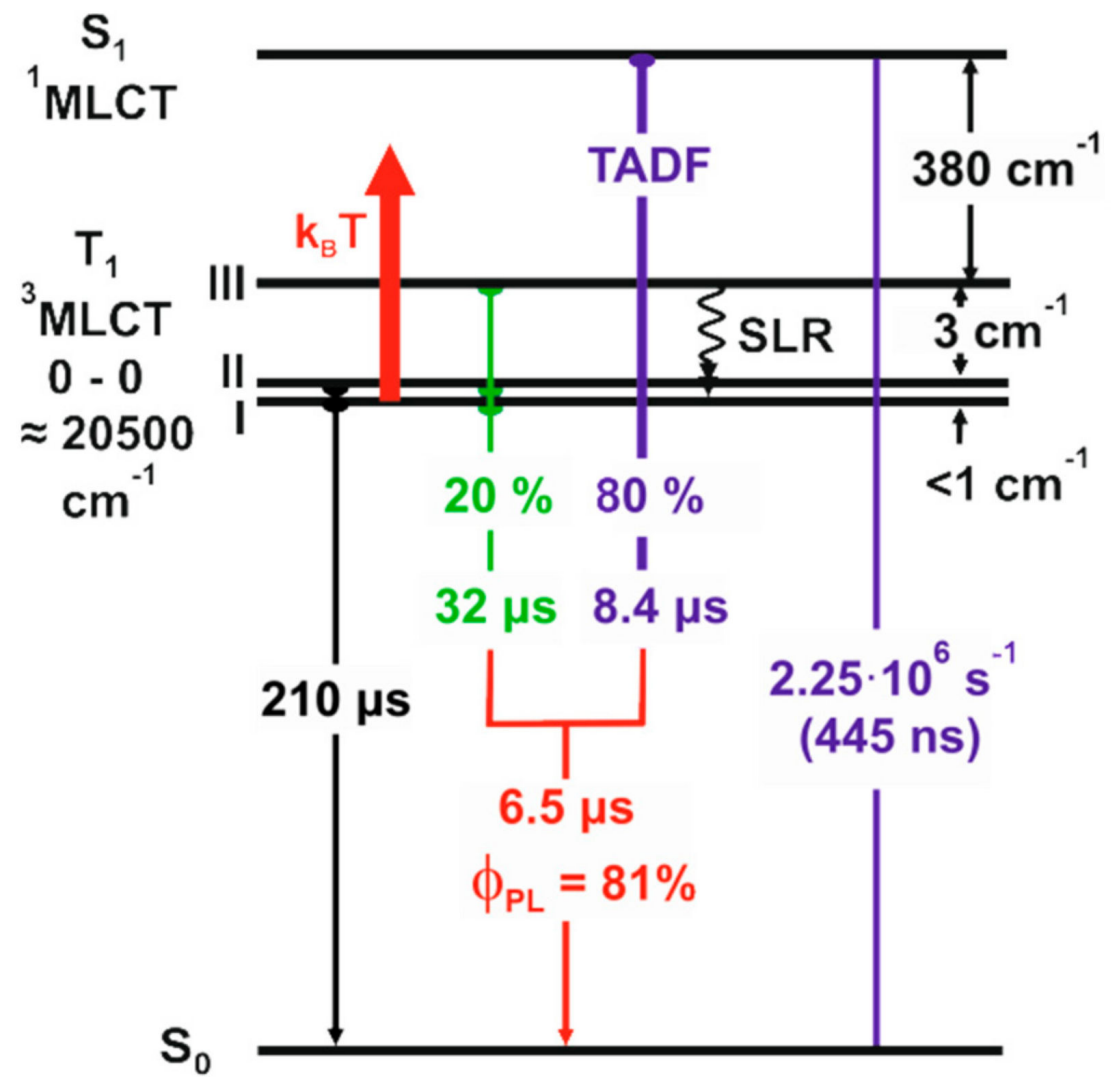
Energy level diagram, decay data and rates for Cu_2_I_2_(Ph_2_Ppy)_3_, **1-I** powder. The T_1_ substates I and II emit independently at T = 1.2 K with τ(I) ≈ τ(II) ≈ 210 µs. The transition rate k(III→S_0_) amounts to 8.3 × 10^4^ s^−1^, corresponding to τ(III) = 12 µs. The spin-lattice relaxation time at T = 1.2 K is determined to τ(SLR) = 59 µs. The T_1_→S_0_(0-0) energy is estimated from the blue energy flank of the phosphorescence band as displayed in Figure 5. At T = 300 K, the total emission is composed of phosphorescence (20%) and TADF (80%), leading to a combined decay time of 6.5 µs at Φ_PL_ = 81%.

If we assume constant Φ_PL_ over the whole temperature range, as approximately justified (Table 4), very good fit of Equation (1) to the measured decay times is realized (Figure 7). As photophysical fit parameters we find ∆E(II–I) ≈ 0 cm^−1^ (meaning < 1 cm^−1^) and ∆E(III–I) = 3 cm^−1^ (0.37 meV). These values are smaller than the ones obtained from our SOC-TDDFT computations, but the splitting pattern is well reproduced (Table 3). For the manifold of the three triplet substates we expect further that for T < 1 K, a low-temperature plateau of the emission decay time τ(T) will be adapted near 210 µs. Although, the individual decay times of the substates I and II cannot be determined directly, we will roughly set τ(I) ≈ τ(II) ≈ 210 µs. This is not unreasonable, since any larger deviation from this approach would not fit to the set of experimental data and the fitting procedure. Moreover, from the fit, we obtain k(III) = 8.3 × 10^4^ s^−1^, corresponding to formally 12 µs (compare Figure 8, below and Table 5). Since the III→S_0_ decay path is much faster than the I,II→S_0_ decays, state III involvement is already of importance at T = 1.2 K by reducing the decay time from ≈ 210 µs to 174 µs as found experimentally (Figure 6).

The short decay component of 10 µs observed at T = 1.2 K (Figure 6) is assigned to the direct mechanism of SLR [117] from substate III to both substates I and II. Using the fit data presented above, we can determine the SLR rate k(SLR) for these processes by

k(SLR) = k(obs) − k(III→S_0_) (2)

Herein, k(obs) corresponds to the observed decay component of 10 µs and k(III→S_0_) is the rate of the III→S_0_ transition with 8.3 × 10^4^ s^−1^. Accordingly, we find k(SLR) = 1.7 × 10^4^ s^−1^ corresponding to τ(SLR) = 59 µs. This means, the SLR process is relatively slow. Values of similar size have, for example, been reported for Ir(III)complexes [125,126].

The fitting procedure gives also ∆E(S_1_-T_1_) = 380 cm^−1^ (47 meV), the energy gap that is crucial for TADF properties. The computed value for the T_1_ state geometry fits relatively well (411 cm^−1^, 51 meV, Table 3). Moreover, the rate of the S_1_→S_0_ fluorescence, also resulting from the fit, amounts to k(S_1_→S_0_) = 2.25 × 10^6^ s^−1^ (445 ns, Figure 7 and Figure 8). However, the corresponding prompt fluorescence decay cannot be measured directly, since the S_1_–T_1_ ISC is orders of magnitude faster. It amounts to around 10 ps [103,104,105,118,119,120,121] as already discussed above. The computations lead to a value of 1/k(S_1_–S_0_) = τ(S_1_–S_0_) = τ(S_1_) = 1.5 µs for the prompt fluorescence (Table 3), thus, being overestimated by a factor of about three. Even the experimental S_1_→S_0_ transition rate is relatively slow for an “allowed” fluorescence, but this is in line with the distinct CT character of the transition. At small HOMO-LUMO overlap, one obtains small exchange interaction and hence, a small ∆E(S_1_-T_1_) gap (as required for TADF materials), however, as well as a slow S_1_→S_0_ transition rate [37,48]. For completeness, it is remarked that design of materials with faster S_1_→S_0_ transition rates and small gaps ∆E(S_1_-T_1_) is highly attractive and indeed, became possible recently [40,41,42,43,44,52].

Interestingly, the emission intensity at ambient temperature represents a combined phosphorescence and TADF. According to the Supporting Information of ref. [87] and [82], we find for the ratio of phosphorescence intensity Int(T_1_) to the total emission intensity Int(tot)

(3) $$\frac{\mathrm{Int}\left(T_{1}\right)}{\mathrm{Int}\left(\mathrm{tot}\right)}=\frac{1}{1+\frac{\tau \left(T_{1}\right)}{3\cdot \tau \left(S_{1}\right)}\cdot \exp\left(-\frac{\Delta E\left(S_{1}-T_{1}\right)}{k_{B}T}\right)}$$

For the phosphorescence decay time τ(T_1_), we can insert the value given by the plateau displayed in Figure 7 with τ(plateau) = 32 µs or we can calculate it from the average decay time determined from the three triplet substates according to [19,117,127].

(4) $$\tau \left(T_{1}\right)=3{\left(\frac{1}{\tau \left(I\right)}+\frac{1}{\tau \left(\mathrm{II}\right)}+\frac{1}{\tau \left(\mathrm{III}\right)}\right)}^{-1}$$

with τ(I) ≈ τ(I) ≈ 210 µs and τ(III) = 12 µs, we also obtain τ(T_1_) = 32 µs, as expected.

Inserting into Equation (3) the mean value of τ(T_1_) = 32 µs, τ(S_1_) = 0.445 µs and ∆E(S_1_-T_1_) = 380 cm^−1^, we find for ambient temperature the percentage of phosphorescence intensity relative to the total emission intensity of ≈20%. Thus, the fractional emission intensity of the TADF-only emission amounts to ≈80%. Accordingly, compound Cu_2_I_2_(Ph_2_Ppy)_3_ **1-I** shows two emission decay paths, as already indicated by the relatively fast phosphorescence rate and by the temperature dependent development of the emission spectra (Figure 5). The rate of the TADF-only process is expressed by

k^r^(TADF-only) = k^r^(com) − k^r^(T_1_) (5)

With the values of the radiative combined rate of k^r^(com) = 1.25 × 10^5^ s^−1^ (k^r^(com) = Φ_PL_/τ = 0.81/(6.5 µs), Table 4) and the radiative phosphorescence rate of k^r^(T_1_) = 2.88 × 10^4^ s^−1^ (τ^r^ = 35 µs, Table 4), we obtain for the radiative TADF-only process k^r^(TADF-only) = 9.62 × 10^4^ s^−1^ corresponding to τ^r^ = 10.4 µs decay time. Hence, the radiative decay time of the combined process, phosphorescence and TADF, is significantly shorter than the TADF-only process. This is valid for the radiative as well as for the measured decays. The combined emission decays by 23 % faster than the TADF-only emission. This is a favorable result and may help in future design strategies to shorten the overall emission decay time of emitter materials to reduce OLED device stability problems [68].

Essential properties of Cu_2_I_2_(Ph_2_Ppy)_3_, **1-I** worked out above are summarized in Table 5 and visualized by an energy level diagram in Figure 8.

## 3. Summarizing Conclusions

In this report, we present an overview over a series of P∩N linked Cu(I) dimers of the type of Cu_2_X_2_(P∩N)_3_ with respect to their X-ray structures and with particular focus on photo-luminescence data. Within the series of eight different compounds, the emission color varies from green to red, the ambient temperature radiative decay time τ^r^ covers a range from 8 to 21 µs and the emission quantum yield Φ_PL_ lies between 9 and 81%. All compounds studied show combined TADF and phosphorescence at ambient temperature. This is due to relatively large spin-orbit coupling (SOC) experienced by the lowest lying triplet state T_1_. Around T = 70 K, the TADF component is largely frozen out and only phosphorescence is observed. The set of data described in the first part, leads us to select a specific compound, namely Cu_2_I_2_(Ph_2_Ppy)_3_ **1-I**, for deeper photophysical characterizations by DFT and SOC-TDDFT computations and for emission measurements over the large temperature range of 1.2 ≤ T ≤ 300 K. Thus, the interplay of phosphorescence and TADF is clarified and properties of the lowest excited singlet and triplet states can be revealed in detail. For example, we determine the S_1_→S_0_ transition rate, the ∆E(S_1_−T_1_) gap, detailed triplet state features, such as the T_1_→S_0_ transition rate as well as the rates from the substates, zero-field splitting (ZFS) of the T_1_ state and the spin-lattice relaxation (SLR) rate between triplet substates. Interestingly, the investigation of the decay time as function of temperature allows us to determine electronic splitting features of the order of less than 1 cm^−1^ (0.1 meV), although the spectral halfwidth of about 3500 cm^−1^ (0.43 eV) is by a factor of 3500 larger.

Thus, this presentation proceeds from an overview over properties of a series of related Cu(I) dimers to detailed photophysical characterization at the state of art. Moreover, it becomes evident that the occurrence of a combined emission process at ambient temperature, consisting of distinct phosphorescence and TADF, or if applied in an OLED, consisting of combined singlet and triplet harvesting, leads to significant shortening of the overall photoluminescent decay time. This is a favorable result and may help in future design to improve the performance of OLED devices with respect to decrease of roll-off and stability problems and even increase of the external quantum efficiency (EQE).

## 4. Materials and Methods

### 4.1. General

All commercially available solvents and starting materials were used without further purification. 2-(Diphenylphosphino)pyridine was purchased from Acros Organics. 2-(Diphenylphosphino)pyrimidine, **2**,1-(diphenylphosphino)isoquinoline, **3**, [97] complexes **1-X** (X = Cl, Br, I) and **3-I** were prepared according to described procedures [34]. Elemental analyses were carried out by the Center for Chemical Analysis of the Faculty of Natural Sciences of the University Regensburg. Single-crystal structure analysis was carried out on a Bruker Smart X2S (**1-Br**), Bruker X8 APEX-II (**2-I**) and STOE-IPDS (**3-I**) diffractometer with graphite-monochromated Mo-Kα radiation (λ = 0.71073 Å). The structures were solved by direct methods (SHELXS-97 [128,129], SIR-92 [130]) and refined by full-matrix least-squares on F^2^ (SHELXL-97 [131,132] and SHELXL-2014/7 [133]). The H atoms were calculated geometrically and a riding model was applied in the refinement process. Crystallographic details can be found in Table 6.

### 4.2. Photophysical Measurements

The complexes were investigated as powders. Emission spectra and decay curves were measured by use of a Fluorolog 3 spectrometer (Horiba Jobin Yvon, Munich, Germany) equipped with a cooled photomultiplier tube. The spectra were corrected with respect to the wavelength dependence of the instrument. The decay behavior of the phosphorescence was recorded using a multichannel scaler card (P7887, Fast ComTec, Munich, Germany) with a time resolution of 250 ps. For excitation, the third harmonic of a pulsed Nd:YAG laser (355 nm, pulse width < 8 ns) was used. A Konti IT (CryoVac, Troisdorf, Germany) cryostat was applied for the variation of temperature between 1.2 K and 300 K. Quantum yield measurements at ambient temperature and at 77 K were carried out with an integrating sphere applying a C9920-02 system (Hamamatsu, Herrsching am Ammersee, Germany).

### 4.3. Computational Investigations

Density functional theory (DFT) geometry optimizations were performed with the Turbomole [134] package using a basis set of triple-zeta plus polarization (def2-TZVP) quality [135] and the hybrid B3LYP exchange-correlation functional [136]. For iodine the corresponding effective core potential [137] was employed. Time-dependent DFT (TDDFT) calculations were carried out with ADF2014 [101] as mentioned in the main text.

### 4.4. Syntheses

General Procedure for the syntheses of complexes **2-X** and **3-Br** according to a simplified literature method: The copper(I) halide salt (2 equivalents) and the ligand (3 equivalents) were suspended in dichloromethane (15 mL) and stirred 12 h under ambient conditions. To the filtered reaction solution diethyl ether was added. The formed solid was filtered off, washed with diethyl ether and dried in vacuum. As soon as the complexes were precipitated form the reaction mixture, they were not sufficiently soluble in common weakly or non-coordinating solvents to perform NMR-spectroscopy. Slow gas phase diffusion of diethyl ether into a solution which was obtained by filtration of the crude reaction mixture gave single crystal suitable for X-ray diffraction.

*[(2-Diphenylphosphino)pyrimidine)_3_Cu_2_Cl_2_]* (**2-Cl**). **2** (150 mg, 0.57 mmol), CuCl (38 mg, 0.38 mmol). Yield: 127 mg, 0.128 mmol, 68%, yellow powder. Anal. Calcd for C_48_H_39_Cu_2_Cl_2_N_6_P_3_ (990.79 g·mol^−1^): C, 58.19; H, 3.97; N, 8.48. Found: C, 58.45; H, 4.12; N, 8.43.

*[(2-Diphenylphosphino)pyrimidine)_3_Cu_2_Br_2_]* (**2-Br**): **2** (150 mg, 0.57 mmol) CuBr (54 mg, 0.38 mmol). Yield: 156 mg, 0.144 mmol, 74%, yellow powder. Anal. Calcd for C_48_H_39_Cu_2_Br_2_N_6_P_3_ (1079.70 g·mol^−1^): C, 53.40; H, 3.64; N, 7.78. Found: C, 53.24; H, 3.53; N, 7.76.

*[(2-Diphenylphosphino)pyrimidine)_3_Cu_2_I_2_]* (**2-I**): **2** (150 mg, 0.57 mmol) CuI (72 mg, 0.38 mmol). Yield: 176 mg, 0.150 mmol, 79%, yellow powder. Anal. Calcd for C_48_H_39_Cu_2_I_2_N_6_P_3_·½CH_2_Cl_2_ (1173.69 g·mol^−1^): C, 47.90; H, 3.32; N, 6.91. Found: C, 47.73; H, 3.44; N, 6.75.

*[(2-Diphenylphosphino)isoquinoline)_3_Cu_2_Br_2_]* (**3-Br**). **3** (75 mg, 0.24 mmol) CuBr (23 mg, 0.16 mmol). Yield: 72 mg, 0.059 mmol, 75%, yellow powder. Anal. Calcd for C_63_H_48_Cu_2_Br_2_N_3_P_3_·½CH_2_Cl_2_ (1205.84 g·mol^−1^): C, 60.08; H, 3.89; N, 3.31. Found: C, 60.29; H, 4.08; N, 3.23.

## Figures and Tables

**Table 1:** Selected bond lengths (Å) and bond angles (°) for Cu_2_X_2_(Ph_2_Ppy)_3_, **1-X** (with X = Cl, Br, I), Cu_2_I_2_(Ph_2_Ppym)_3_, **2-I** and Cu_2_I_2_(Ph_2_Piqn)_3_, **3-I**.

Compound	1-Cl ^a^	1-Br	1-I ^a^	2-I	3-I·CH_2_Cl_2_	3-I ^a^
Cu1–Cu2	2.878(1)	2.883(1)	2.7694(5)	2.693(1)	2.799(1)	2.7204(6)
Cu1–P1	2.242(1)	2.240(1)	2.2514(6)	2.263(1)	2.292(1)	2.2555(8)
Cu1–P2	2.248(1)	2.258(1)	2.2522(7)	2.249(1)	2.291(1)	2.2404(9)
Cu2–P3	2.224(1)	2.237(1)	2.2507(7)	2.244(1)	2.285(1)	2.2468(9)
Cu2–N	2.106(3)	2.098(4)	2.104(1)	2.101(3)	2.140(3)	2.076(2)
Cu1–X1	2.395(1)	2.570(1)	2.6733(7)	2.684(1)	2.699(1)	2.6930(5)
Cu1–X2	2.426 (1)	2.522(1)	2.6803(5)	2.718(1)	2.641(1)	2.6954(5)
Cu2–X1	2.436(1)	2.509(1)	2.7280(6)	2.647(1)	2.714(1)	2.6277(5)
Cu2–X2	2.390(1)	2.543(1)	2.6446(5)	2.702(1)	2.687(1)	2.6802(5)
X1–Cu1–X2	98.38(4)	101.89(3)	107.63(2)	106.85(2)	107.02(2)	108.86(2)
X1–Cu2–X2	98.25(3)	103.00(3)	107.07(1)	108.41(2)	105.31(2)	111.32(2)
N–Cu2–P3	123.23(8)	123.35(9)	117.47(2)	115.9(1)	111.8(1)	120.35(2)
P1–Cu1–P2	123.80(4)	123.90(5)	119.87(2)	118.40(4)	118.8(1)	118.16(3)
Cu1–X1–Cu2	73.42(3)	69.17(2)	61.68(1)	60.69(1)	62.27(2)	61.48(1)
Cu1–X2–Cu2	73.13(3)	69.40(2)	62.67(1)	59.59(1)	63.38(2)	60.80(1)

^a^—values from Ref. [34].

**Table 2:** Selected bond lengths [Å] and angles [°] for Cu_2_I_2_(Ph_2_Ppy)_3_ **1-I** as obtained from DFT calculations (B3LYP/TZVP) for the S_0_ and T_1_ minimum compared to the values from the X-ray structure determination. The atom numbering scheme is analogous to Cu_2_Br_2_(Ph_2_Ppy)_3_ **1-Br** shown in Figure 1.

	Exp ^a^	Calculations Geometry S_0_	T_1_
Cu1–Cu2	2.77	2.86	2.59
Cu2–I1	2.73	2.71	2.67
Cu1–I1	2.67	2.76	2.76
Cu1–P2	2.25	2.36	2.36
Cu1–P1	2.25	2.36	2.35
Cu2–P3	2.25	2.33	2.37
Cu2–N1	2.10	2.22	2.03
I1–Cu1–I2	107.6	106.8	102.5
I1–Cu2–I2	107.1	108.9	103.4
Cu1–I1–Cu2	61.7	63.2	56.9
Cu1–I2–Cu2	62.7	62.3	55.9
P1–Cu1–Cu2	87.1	85.5	85.0
P2–Cu1–P1	119.9	119.7	117.3

^a^—taken from ref. [34].

**Table 3:** SOC-TDDFT (B3LYP/DZP) vertical excitation energies [eV] and radiative lifetime calculated for 100% quantum yield in µs (in brackets) for the S_1_ state and the three substates of the T_1_ state of Cu_2_I_2_(Ph_2_Ppy)_3_, **1-I**. Singlet-triplet splittings Δ(S_1_−T_1_) [cm^−1^] obtained from the average triplet energy and zero field splittings ZFS [cm^−1^] are also given. Results are presented for calculations at the S_0_ optimized geometry with and without solvent effects and at the T_1_ geometry in gas phase.

Geometry	I(T_1_)	II(T_1_)	III(T_1_)	S_1_	Δ(S_1_−T_1_)	ZFS
						∆E(III−I)
S_0_	2.418 (752.4)	2.420 (95.1)	2.424 (6.5)	2.437 (1.6)	129	52
S_0_(solvent)	2.796 (92.5)	2.797 (8.1)	2.801 (1.3)	2.816 (0.2)	144	39
T_1_	1.452 (>10^3^)	1.453 (>10^3^)	1.456 (54.4)	1.507 (2.8)	411	32
Exp ^a^	2.54 (≈210)	2.54 (≈210)	2.54 (12)	≈2.588 ^b^ (0.445)	380	3

^a^—compare Figure 8, below. ^b^—estimated from the blue flank of the emission spectrum at T = 1.3 K + Δ(S1-T1) = 380 cm^−1^ (Figure 5, below).

**Table 4:** Luminescence data of a series of P∩N linked Cu(I) dimer complexes.

Compound		λ_max_(300 K) ^a^ [nm]	Φ_PL_(300 K) ^b^ [%]	τ(300 K) ^a^ [µs]	*k*^r^(300 K) ^c^ [s^−1^]	*k*^nr^(300K) ^d^ [s^−1^]	λ_max_(77 K) ^a^ [nm]	Φ_PL_(77 K) ^b^ [%]	τ(77 K) ^a^ [µs]	*k*^r^(77 K) ^c^ [s^−1^]	*k*^nr^(77 K) ^d^ [s^−1^]
Cu_2_Cl_2_(Ph_2_Ppy)_3_	**1-Cl**	577	37	7.9	4.7 × 10^4^	8.0 × 10^4^	592	71	65	1.1 × 10^4^	4.5 × 10^3^
Cu_2_Br_2_(Ph_2_Ppy)_3_	**1-Br**	545	53	8.8	6.0 × 10^4^	5.3 × 10^4^	567	89	110	8.1 × 10^3^	1.0 × 10^3^
Cu_2_I_2_(Ph_2_Ppy)_3_	**1-I**	539	81	6.5	1.25 × 10^5^	2.92 × 10^4^	552	92	32	2.88 × 10^4^	2.5 × 10^3^
Cu_2_Cl_2_(Ph_2_Ppym)_3_	**2-Cl**	616	9	1.2	7.5 × 10^4^	7.6 × 10^5^	626	14	30	4.7 × 10^3^	2.9 × 10^4^
Cu_2_Br_2_(Ph_2_Ppym)_3_	**2-Br**	583	33	2.5	1.3 × 10^5^	2.7 × 10^5^	584	56	29	1.9 × 10^4^	1.5 × 10^4^
Cu_2_I_2_(Ph_2_Ppym)_3_	**2-I**	565	13	1.7 ^e^ (2.7 ^f^)	7.6 × 10^4^	5.1 × 10^5^	575	67	17.4 ^e^	3.9 × 10^4^	1.9 × 10^4^
Cu_2_Br_2_(Ph_2_Piqn)_3_	**3-Br**	660	11	2.0 ^e^	5.5 × 10^4^	4.5 × 10^5^	668	24	42 ^e^	5.7 × 10^3^	1.8 × 10^4^
Cu_2_I_2_(Ph_2_Piqn)_3_	**3-I**	636	38	3.3 ^e^	1.2 × 10^5^	1.9 × 10^5^	645	59	22	2.7 × 10^4^	1.9 × 10^4^

^a^—excitation wavelength λ_exc_ = 372 nm; ^b^—accuracy at 77 K: ± 10%, at 300 K: ± 5 % (relative error), (excitation wavelength λ_exc_ = 400 nm); ^c^—determined according to k^r^ = Φ_PL_/τ; ^d^—determined according to k^nr^ = (1-Φ_PL_)/τ; ^e^—the decay curve deviates from mono-exponential behavior. The lifetime given represents the main component; ^f^—long component.

**Table 5:** Energies, emission quantum yields and decay data of Cu_2_I_2_(Ph_2_Ppy)_3_, **1-I** (powder). Note the different temperatures.

Property	Value
E_0-0_(T_1_–S_0_) ^a^	20,500 cm^−1^
	2.541 eV
E_0-0_(S_1_–S_0_) ^b^	20,880 cm^−1^
	2.588 eV
Φ_PL_(300 K)	81%
Φ_PL_(77 K)	92%
k(S_1_–S_0_)	2.25 × 10^6^ s^−1^
	(445 ns)
k(T_1_–S_0_), plateau	3.1 × 10^4^ s^−1^
	(32 µs)
k^r^(T_1_–S_0_)	2.88 × 10^4^ s^−1^
	(35 µs)
k(TADF + phos), 300 K observed	15.4 × 10^4^ s^−1^
	6.5 µs
k(TADF-only), 300 K	11.9 × 10^4^ s^−1^
	(8.4 µs)
∆E(S_1_–T_1_)	380 cm^−1^
	(47 meV)
∆E(II–I)	<1 cm^−1^
∆E(III–I,II)	3 cm^−1^
	(0.37 meV)
k(III–S_0_)	8.3 × 10^4^ s^−1^
	(12 µs)
k(I–S_0_) ≈ k(II–S_0_)	≈4.76 × 10^3^ s^−1^
	(≈210 µs)
k(SLR) ^c^ at 1.2 K	1.7 × 10^4^ s^−1^
	(59 μs)

^a^—estimated from the blue flank of the emission spectrum at T = 1.3 (Figure 5); ^b^—E_0-0_(T_1_–S_0_) + ∆E(S_1_-T_1_); ^c^—spin-lattice relaxation rate between the triplet substates III and I, II at T = 1.2 K.

**Table 6:** Crystal data and data collection and structure refinement details for **1-Br**, **2-I** and **3-I**.

	1-Br	2-I	3-I·CH_2_Cl_2_
Empirical formula	C_51_H_42_Br_2_Cu_2_N_3_P_3_	C_48_H_39_Cu_2_I_2_N_6_P_3_·CH_2_Cl_2_	C_63_H_48_Cu_2_I_2_N_3_P_3_·CH_2_Cl_2_
*M*_r_, g mol^−1^	1076.69	1258.57	1405.78
Size, mm^3^	0.40 × 0.25 × 0.11	0.28 × 0.18 × 0.08	0.20 × 0.18 × 0.15
Crystal system	monoclinic	monoclinic	triclinic
Space group	*P*2_1_/n	*C*2/*c*	$P\bar{1}$
*a*, Å	14.202(2)	42.494(5)	12.4000(13)
*b*, Å	18.139(3)	11.0172(14)	15.5224(17)
*c*, Å	17.628(3)	22.311(3)	16.3296(17)
*α*, deg	90	90	97.462(12)
*β*, deg	98.223(5)	103.076(3)	106.923(12)
*γ*, deg	90	90	92.752(13)
*V*, Å^3^	4494.5(11)	10174(2)	2969.3(6)
*ρ*_calcd._, g cm^−1^	1.591	1.643	1.572
*Z*	4	8	2
μ(Mo-K_α_), mm^−1^	2.87	2.29	1.97
T, K	300	293	123
Θ range, deg	1.6–25.1	3.0–28.5	2.2–27.0
Measured reflections	27,865	14,027	42,450
Independent reflections	7896	9516	11,932
Reflections with I > 2σ(I))	5151	7611	7253
Absorption correction	multi-scan	multi-scan	analytical
T_min_/T_max_	0.39, 0.74	0.894, 0.914	0.725, 0.769
Restraints/refined param.	0/550	0/617	0/685
R1 (I ≥ 2σ(I))	0.047	0.031	0.034
wR2	0.092	0.083	0.074
ρ_fin_ (max/min), e Å^−3^	1.15/−0.65	0.60/−0.54	1.81/−1.45
CCDC no.	2,034,780	2,034,779	2,034,781

## Data Availability

CCDC 2034780 (**1-Br**), 2034779 (**2-I**), 2034781 (**3-I·CH_2_Cl_2_**), contain supplementary crystallographic data for this paper. This information can be obtained free of charge via https://www.ccdc.cam.ac.uk/structures/.

## Sample Availability

Samples of the compounds are available from the authors.

## References

[1] Helfrich W., Schneider W.G. (1966). Transients of volume-controlled current and of recombination radiation in anthracene. J. Chem. Phys..

[2] Yersin H. (2008). Highly Efficient OLEDs with Phosphorescent Materials.

[3] Brütting W., Adachi C. (2012). Physics of Organic Semiconductors.

[4] Baldo M.A., O’Brien D.F., You Y., Shoustikov A., Sibley S., Thompson M.E., Forrest S.R. (1998). Highly efficient phosphorescent emission from organic electroluminescent devices. Nature.

[5] Adachi C., Baldo M.A., Thompson M.E., Forrest S.R. (2001). Nearly 100% internal phosphorescence efficiency in an organic light-emitting device. J. Appl. Phys..

[6] Yersin H. (2004). Triplet Emitters for OLED applications. Mechanisms of exciton trapping and control of emission properties. Top. Curr. Chem..

[7] Yersin H. (2019). Highly Efficient OLEDs: Materials Based on Thermally Activated Delayed Fluorescence.

[8] Yersin H., Monkowius U. (2010). Komplexe mit Kleinen Singulett-Triplett-Energie-Abständen zur Verwendung in Opto-Elektronischen Bauteilen (Singulett-Harvesting-Effekt). German Patent.

[9] Czerwieniec R., Yu J., Yersin H. (2011). Blue-light emission of Cu(I) complexes and singlet harvesting. Inorg. Chem..

[10] Deaton J.C., Switalski S.C., Kondakov D.Y., Young R.H., Pawlik T.D., Giesen D.J., Harkins S.B., Miller A.J.M., Mickenberg S.F., Peters J.C. (2010). E-type delayed fluorescence of a phosphine-supported Cu_2_(μ-NAr_2_)_2_ diamond core: Harvesting singlet and triplet excitons in OLEDs. J. Am. Chem. Soc..

[11] Zink D.M., Volz D., Baumann T., Mydlak M., Flügge H., Friedrichs J., Nieger M., Bräse S. (2013). Heteroleptic, dinuclear copper (I) complexes for application in organic light-emitting diodes. Chem. Mater..

[12] Dumur F. (2015). Recent advances in organic light-emitting devices comprising copper complexes: A realistic approach for low-cost and highly emissive devices?. Org. Electron..

[13] Lamansky S., Djurovich P., Murphy D., Abdel-Razzaq F., Lee H.E., Adachi C., Burrows P.E., Forrest S.R., Thompson M.E. (2001). Highly phosphorescent bis-cyclometalated iridium complexes: Synthesis, photophysical characterization, and use in organic light emitting diodes. J. Am. Chem. Soc..

[14] Zysman-Colman E. (2017). Iridium (III) in Optoelectronic and Photonics Applications.

[15] Deaton J.C., Castellano F.N., Zysman-Colman E. (2017). Archetypal iridium (III) compounds for optoelectronic and photonic applications. Iridium(III) in Optoelectronic and Photonics Applications.

[16] Tang M.-C., Chan A.K.-W., Chan M.-Y., Yam V.W.-W. (2016). Platinum and gold complexes for OLEDs. Top. Curr. Chem..

[17] Li K., Ming Tong G.S., Wan Q., Cheng G., Tong W.-Y., Ang W.-H., Kwong W.-L., Che C.-M. (2016). Highly phosphorescent platinum (II) emitters: Photophysics, materials and biological applications. Chem. Sci..

[18] Armaroli N., Bolink H.J. (2017). Photoluminescent Materials and Electroluminescent Devices.

[19] Yersin H., Rausch A.F., Czerwieniec R., Hofbeck T., Fischer T. (2011). The triplet state of organo-transition metal compounds. Triplet harvesting and singlet harvesting for efficient OLEDs. Coord. Chem. Rev..

[20] Yersin H., Rausch A.F., Czerwieniec R., Brütting W., Adachi C. (2012). Organometallic emitters for OLEDs: Triplet harvesting, singlet harvesting, case structures, and trends. Physics of Organic Semiconductors.

[21] Yersin H., Finkenzeller W.J., Yersin H. (2007). Triplet emitters for organic light-emitting diodes: Basic properties. Highly Efficient OLEDs with Phosphorescent Materials.

[22] Che C.-M., Kwok C.-C., Lai S.-W., Rausch A.F., Finkenzeller W.J., Zhu N., Yersin H. (2010). Photophysical properties and OLED applications of phosphorescent platinum (II) Schiff base complexes. Chem. Eur. J..

[23] Lin Y.-Y., Chan S.-C., Chan M.C.W., Hou Y.-J., Zhu N., Che C.-M., Liu Y., Wang Y. (2003). Structural, photophysical, and electrophosphorescent properties of platinum (II) complexes supported by tetradentate N_2_O_2_ chelates. Chem. Eur. J..

[24] Cheng G., Kui S.C.F., Ang W.-H., Ko M.-Y., Chow P.-K., Kwong C.-L., Kwok C.-C., Ma C., Guan X., Low K.-H. (2014). Structurally robust phosphorescent [Pt(O^N^C^N)] emitters for high performance organic light-emitting devices with power efficiency up to 126 lm W^−1^ and external quantum efficiency over 20%. Chem. Sci..

[25] Mao M., Peng J., Lam T.-L., Ang W.-H., Li H., Cheng G., Che C.-M. (2019). High-performance organic light-emitting diodes with low-efficiency roll-off using bulky tetradentate [Pt(O^N^C^N)] emitters. J. Mater. Chem. C.

[26] Cheng G., Kwak Y., To W.-P., Lam T.-L., Tong G.S.M., Sit M.-K., Gong S., Choi B., Choi W.I., Yang C. (2019). High-efficiency solution-processed organic light-emitting diodes with tetradentate platinum(II) emitters. ACS Appl. Mater. Interfaces.

[27] Vezzu D.A.K., Deaton J.C., Jones J.S., Bartolotti L., Harris C.F., Marchetti A.P., Kondakova M., Pike R.D., Huo S. (2010). Highly luminescent tetradentate bis-cyclometalated platinum complexes: Design, synthesis, structure, photophysics, and electroluminescence application. Inorg. Chem..

[28] Fleetham T., Li G., Wen L., Li J. (2014). Efficient “pure” blue OLEDs employing tetradentate Pt complexes with a narrow spectral bandwidth. Adv. Mater..

[29] Czerwieniec R., Yersin H. (2015). Diversity of copper(I) complexes showing thermally activated delayed fluorescence: Basic photophysical analysis. Inorg. Chem..

[30] Osawa M., Hoshino M., Hashimoto M., Kawata I., Igawa S., Yashima M. (2015). Application of three-coordinate copper(I) complexes with halide ligands in organic light-emitting diodes that exhibit delayed fluorescence. Dalton Trans..

[31] Yu R., Lu C.-Z., Yersin H. (2018). Ionic [Cu(NN)(PP)]^+^ TAD9727 F complexes with pyridine-based diimine chelating ligands and their use in OLEDs. Highly Efficient OLEDs.

[32] Hirtenlehner C., Monkowius U. (2012). Syntheses, crystal structures and blue luminescence of Cu_2_X_2_(Ph_3_P)_2_[(−)-nicotine]_2_ (X. = Br, I). Inorg. Chem. Commun..

[33] So G.K.-M., Cheng G., Wang J., Chang X., Kwok C.-C., Zhang H., Che C.-M. (2017). Efficient color-tunable copper(I) complexes and their applications in solution-processed organic light-emitting diodes. Chem. Asian J..

[34] Zink D.M., Bächle M., Baumann T., Nieger M., Kühn M., Wang C., Klopper W., Monkowius U., Hofbeck T., Yersin H. (2013). Synthesis, structure, and characterization of dinuclear copper(I) halide complexes with P^N ligands featuring exciting photoluminescence properties. Inorg. Chem..

[35] Wallesch M., Volz D., Zink D.M., Schepers U., Nieger M., Baumann T., Bräse S. (2014). Bright coppertunities: Multinuclear Cu(I) complexes with N-P ligands and their applications. Chem. Eur. J..

[36] Leitl M.J., Zink D.M., Schinabeck A., Baumann T., Volz D., Yersin H. (2016). Copper(I) complexes for thermally activated delayed fluorescence: From photophysical to device properties. Top. Curr. Chem..

[37] Czerwieniec R., Leitl M.J., Homeier H.H.H., Yersin H. (2016). Cu(I) complexes–thermally activated delayed fluorescence. Photophysical approach and material design. Coord. Chem. Rev..

[38] Hamze R., Peltier J.L., Sylvinson D., Jung M., Cardenas J., Haiges R., Soleilhavoup M., Jazzar R., Djurovich P.I., Bertrand G. (2019). Eliminating nonradiative decay in Cu(I) emitters: 99% quantum efficiency and microsecond lifetime. Science.

[39] Hamze R., Shi S., Kapper S.C., Muthiah Ravinson D.S., Estergreen L., Jung M.-C., Tadle A.C., Haiges R., Djurovich P.I., Peltier J.L. (2019). “Quick-silver>” from a systematic study of highly luminescent, two-coordinate, d^10^ coinage metal complexes. J. Am. Chem. Soc..

[40] Shi S., Jung M.C., Coburn C., Tadle A., Sylvinson M.R.D., Djurovich P.I., Forrest S.R., Thompson M.E. (2019). Highly efficient photo- and electroluminescence from two-coordinate Cu(I) complexes featuring nonconventional N-heterocyclic carbenes. J. Am. Chem. Soc..

[41] Di D., Romanov A.S., Yang L., Richter J.M., Rivett J.P., Jones S., Thomas T.H., Jalebi M.A., Friend R.H., Linnolahti M. (2017). High-performance light-emitting diodes based on carbene-metal-amides. Science.

[42] Conaghan P.J., Menke S.M., Romanov A.S., Jones S.T.E., Pearson A.J., Evans E.W., Bochmann M., Greenham N.C., Credgington D. (2018). Efficient vacuum-processed light-emitting diodes based on carbene-metal-amides. Adv. Mater..

[43] Romanov A.S., Jones S.T.E., Gu Q., Conaghan P.J., Drummond B.H., Feng J., Chotard F., Buizza L., Foley M., Linnolahti M. (2020). Carbene metal amide photoemitters: Tailoring conformationally flexible amides for full color range emissions including white-emitting OLED. Chem. Sci..

[44] Föller J., Ganter C., Steffen A., Marian C.M. (2019). Computer-aided design of luminescent linear N-heterocyclic carbene copper(I) pyridine complexes. Inorg. Chem..

[45] Osawa M., Hoshino M., Yersin H. (2018). Molecular design and synthesis of metal complexes as emitters for TADF-type OLEDs. Highly Efficient OLEDs.

[46] Nozaki K., Iwamura M., Yersin H. (2018). Highly emissive d^10^ metal complexes as TADF emitters with versatile structures and photophysical properties. Highly Efficient OLEDs.

[47] Tsuboyama A., Yersin H. (2018). Luminescent dinuclear copper(I) complexes with short intramolecular Cu-Cu distances. Highly Efficient OLEDs.

[48] Yersin H., Czerwieniec R., Shafikov M.Z., Suleymanova A.F. (2017). TADF material design: Photophysical background and case studies focusing on CuI and AgI complexes. Chemphyschem.

[49] Armaroli N., Accorsi G., Cardinali F., Listorti A., Balzani V., Campagna S. (2007). Photochemistry and photophysics of coordination compounds: Copper. Photochemistry and Photophysics of Coordination Compounds I.

[50] Schinabeck A., Leitl M.J., Yersin H. (2018). Dinuclear Cu(I) complex with combined bright TADF and phosphorescence. Zero-field splitting and spin-lattice relaxation effects of the triplet state. J. Phys. Chem. Lett..

[51] Schinabeck A., Rau N., Klein M., Sundermeyer J., Yersin H. (2018). Deep blue emitting Cu(I) tripod complexes. Design of high quantum yield materials showing TADF-assisted phosphorescence. Dalton Trans..

[52] Schinabeck A., Chen J., Kang L., Teng T., Homeier H.H.H., Suleymanova A.F., Shafikov M.Z., Yu R., Lu C.-Z., Yersin H. (2019). Symmetry-based design strategy for unprecedentedly fast decaying thermally activated delayed fluorescence (TADF). Application to dinuclear Cu(I) compounds. Chem. Mater..

[53] Xu K., Chen B.-L., Yang F., Liu L., Zhong X.-X., Wang L., Zhu X.-J., Li F.-B., Wong W.-Y., Qin H.-M. (2021). Largely color-tuning prompt and delayed fluorescence: Dinuclear Cu(I) halide complexes with tert -amines and phosphines. Inorg. Chem..

[54] Gan X.-M., Yu R., Chen X.-L., Yang M., Lin L., Wu X.-Y., Lu C.-Z. (2018). A unique tetranuclear Ag(I) complex emitting efficient thermally activated delayed fluorescence with a remarkably short decay time. Dalton Trans..

[55] Shafikov M.Z., Suleymanova A.F., Czerwieniec R., Yersin H. (2017). Design strategy for Ag(I)-based thermally activated delayed fluorescence reaching an efficiency breakthrough. Chem. Mater..

[56] Shafikov M.Z., Suleymanova A.F., Schinabeck A., Yersin H. (2018). Dinuclear Ag(I) complex designed for highly efficient thermally activated delayed fluorescence. J. Phys. Chem. Lett..

[57] Osawa M., Kawata I., Ishii R., Igawa S., Hashimoto M., Hoshino M. (2013). Application of neutral d^10^ coinage metal complexes with an anionic bidentate ligand in delayed fluorescence-type organic light-emitting diodes. J. Mater. Chem. C.

[58] Chen J., Teng T., Kang L., Chen X.-L., Wu X.-Y., Yu R., Lu C.-Z. (2016). Highly efficient thermally activated delayed fluorescence in dinuclear Ag(I) complexes with a bis-bidentate tetraphosphane bridging ligand. Inorg. Chem..

[59] Chan K.-T., Lam T.-L., Yu D., Du L., Phillips D.L., Kwong C.-L., Tong G.S.M., Cheng G., Che C.-M. (2019). Strongly luminescent tungsten emitters with emission quantum yields of up to 84 %: TADF and high-efficiency molecular tungsten OLEDs. Angew. Chem. Int. Ed. Engl..

[60] Fernandez-Cestau J., Bertrand B., Blaya M., Jones G.A., Penfold T.J., Bochmann M. (2015). Synthesis and luminescence modulation of pyrazine-based gold(III) pincer complexes. Chem. Commun..

[61] Zhou D., To W.-P., Kwak Y., Cho Y., Cheng G., Tong G.S.M., Che C.-M. (2019). Thermally stable donor-acceptor type (Alkynyl)gold(III) TADF emitters achieved EQEs and luminance of up to 23.4% and 70 300 cd m-2 in vacuum-deposited OLEDs. Adv. Sci..

[62] Zhou D., To W.-P., Tong G.S.M., Cheng G., Du L., Phillips D.L., Che C.-M. (2020). Tetradentate gold(III) complexes as thermally activated delayed fluorescence (TADF) emitters: Microwave-assisted synthesis and high-performance OLEDs with long operational lifetime. Angew. Chem. Int. Ed. Engl..

[63] Li L.-K., Tang M.-C., Lai S.-L., Ng M., Kwok W.-K., Chan M.-Y., Yam V.W.-W. (2019). Strategies towards rational design of gold(III) complexes for high-performance organic light-emitting devices. Nat. Photonics.

[64] Lee C.-H., Tang M.-C., Kong F.K.-W., Cheung W.-L., Ng M., Chan M.-Y., Yam V.W.-W. (2020). Isomeric tetradentate ligand-containing cyclometalated gold(III) complexes. J. Am. Chem. Soc..

[65] Sakai Y., Sagara Y., Nomura H., Nakamura N., Suzuki Y., Miyazaki H., Adachi C. (2015). Zinc complexes exhibiting highly efficient thermally activated delayed fluorescence and their application to organic light-emitting diodes. Chem. Commun..

[66] Uoyama H., Goushi K., Shizu K., Nomura H., Adachi C. (2012). Highly efficient organic light-emitting diodes from delayed fluorescence. Nature.

[67] Hosokai T., Matsuzaki H., Nakanotani H., Tokumaru K., Tsutsui T., Furube A., Nasu K., Nomura H., Yahiro M., Adachi C. (2017). Evidence and mechanism of efficient thermally activated delayed fluorescence promoted by delocalized excited states. Sci. Adv..

[68] Noda H., Nakanotani H., Adachi C. (2018). Excited state engineering for efficient reverse intersystem crossing. Sci. Adv..

[69] Li X., Shi Y.-Z., Wang K., Zhang M., Zheng C.-J., Sun D.-M., Dai G.-L., Fan X.-C., Wang D.-Q., Liu W. (2019). Thermally activated delayed fluorescence carbonyl derivatives for organic light-emitting diodes with extremely narrow full width at half-maximum. ACS Appl. Mater. Interfaces.

[70] Liu Y., Li C., Ren Z., Yan S., Bryce M.R. (2018). All-organic thermally activated delayed fluorescence materials for organic light-emitting diodes. Nat. Rev. Mater..

[71] Komatsu R., Ohsawa T., Sasabe H., Nakao K., Hayasaka Y., Kido J. (2017). Manipulating the electronic excited state energies of pyrimidine-based thermally activated delayed fluorescence emitters to realize efficient deep-blue emission. ACS Appl. Mater. Interfaces.

[72] Li M., Liu Y., Duan R., Wei X., Yi Y., Wang Y., Chen C.-F. (2017). Aromatic-imide-based thermally activated delayed fluorescence materials for highly efficient organic light-emitting diodes. Angew. Chem. Int. Ed Engl..

[73] Sharma N., Wong M.Y., Samuel I.D.W., Zysman-Colman E., Yersin H. (2018). Solution-processed TADF materials and devices based on organic emitters. Highly Efficient OLEDs.

[74] Wong M.Y., Zysman-Colman E. (2017). Purely organic thermally activated delayed fluorescence materials for organic light-emitting diodes. Adv. Mater..

[75] Chen X.-K., Kim D., Brédas J.-L. (2018). Thermally activated delayed fluorescence (TADF) path toward efficient electroluminescence in purely organic materials: Molecular level insight. Acc. Chem. Res..

[76] Sommer G.A., Mataranga-Popa L.N., Czerwieniec R., Hofbeck T., Homeier H.H.H., Müller T.J.J., Yersin H. (2018). Design of conformationally distorted donor-acceptor dyads showing efficient thermally activated delayed fluorescence. J. Phys. Chem. Lett..

[77] Schmidt T.D., Brütting W., Yersin H. (2018). Efficiency enhancement of organic light-emitting diodes exhibiting delayed fluorescence and nonisotropic emitter orientation. Highly Efficient OLEDs.

[78] Yang Z., Mao Z., Xie Z., Zhang Y., Liu S., Zhao J., Xu J., Chi Z., Aldred M.P. (2017). Recent advances in organic thermally activated delayed fluorescence materials. Chem. Soc. Rev..

[79] Cai X., Su S.-J. (2018). Marching toward highly efficient, pure-blue, and stable thermally activated delayed fluorescent organic light-emitting diodes. Adv. Funct. Mater..

[80] Yersin H., Mataranga-Popa L., Czerwieniec R., Dovbii Y. (2019). Design of a new mechanism beyond thermally activated delayed fluorescence toward fourth generation organic light emitting diodes. Chem. Mater..

[81] Yersin H., Mataranga-Popa L., Li S.-W., Czerwieniec R. (2018). Design strategies for materials showing thermally activated delayed fluorescence and beyond: Towards the fourth-generation OLED mechanism. J. Soc. Inf. Disp..

[82] Leitl M.J., Küchle F.-R., Mayer H.A., Wesemann L., Yersin H. (2013). Brightly blue and green emitting Cu(I) dimers for singlet harvesting in OLEDs. J. Phys. Chem. A.

[83] Gneuß T., Leitl M.J., Finger L.H., Rau N., Yersin H., Sundermeyer J. (2015). A new class of luminescent Cu(I) complexes with tripodal ligands–TADF emitters for the yellow to red color range. Dalton Trans..

[84] Chen X.-L., Yu R., Wu X.-Y., Liang D., Jia J.-H., Lu C.-Z. (2016). A strongly greenish-blue-emitting Cu_4_Cl_4_ cluster with an efficient spin-orbit coupling (SOC): Fast phosphorescence versus thermally activated delayed fluorescence. Chem. Commun..

[85] Bizzarri C., Hundemer F., Busch J., Bräse S. (2018). Triplet emitters versus TADF emitters in OLEDs: A comparative study. Polyhedron.

[86] Baranov A.Y., Berezin A.S., Samsonenko D.G., Mazur A.S., Tolstoy P.M., Plyusnin V.F., Kolesnikov I.E., Artem’ev A.V. (2020). New Cu(I) halide complexes showing TADF combined with room temperature phosphorescence: The balance tuned by halogens. Dalton Trans..

[87] Hofbeck T., Monkowius U., Yersin H. (2015). Highly efficient luminescence of Cu(I) compounds: Thermally activated delayed fluorescence combined with short-lived phosphorescence. J. Am. Chem. Soc..

[88] Li C., Li W., Henwood A.F., Hall D., Cordes D.B., Slawin A.M.Z., Lemaur V., Olivier Y., Samuel I.D.W., Zysman-Colman E. (2020). Luminescent dinuclear Copper(I) complexes bearing an imidazolylpyrimidine bridging ligand. Inorg. Chem..

[89] Hobbollahi E., List M., Hupp B., Mohr F., Berger R.J.F., Steffen A., Monkowius U. (2017). Highly efficient cold-white light emission in a Au_2_CuCl_2_(P∩N)_2_PF_6_ type salt. Dalton Trans..

[90] Yersin H., Fischer T., Monkowius U., Hofbeck T. (2011). Copper Complexes for Optoelectronic Applications. German Patent.

[91] Chen K., Shearer J., Catalano V.J. (2015). Subtle modulation of Cu_4_X_4_L_2_ phosphine cluster cores leads to changes in luminescence. Inorg. Chem..

[92] Artemev A.V., Shafikov M.Z., Schinabeck A., Antonova O.V., Berezin A.S., Bagryanskaya I.Y., Plusnin P.E., Yersin H. (2019). Sky-blue thermally activated delayed fluorescence (TADF) based on Ag(I) complexes: Strong solvation-induced emission enhancement. Inorg. Chem. Front..

[93] Artem’ev A.V., Davydova M.P., Berezin A.S., Ryzhikov M.R., Samsonenko D.G. (2020). Dicopper(I) paddle-wheel complexes with thermally activated delayed fluorescence adjusted by ancillary ligands. Inorg. Chem..

[94] Davydova M.P., Rakhmanova M.I., Bagryanskaya I.Y., Brylev K.A., Artemev A.V. (2020). A 1D coordination polymer based on CuI and 2-(Diphenylphosphino)pyrimidine: Synthesis, structure and luminescent properties. J. Struct. Chem..

[95] Stoïanov A., Gourlaouen C., Vela S., Daniel C. (2018). Luminescent dinuclear Copper(I) complexes as potential thermally activated delayed fluorescence (TADF) emitters: A theoretical study. J. Phys. Chem. A.

[96] Busch J.M., Koshelev D.S., Vashchenko A.A., Fuhr O., Nieger M., Utochnikova V.V., Bräse S. (2021). Various structural design modifications: Para-substituted diphenylphosphinopyridine bridged Cu(I) complexes in organic light-emitting diodes. Inorg. Chem..

[97] Monkowius U., Zabel M., Fleck M., Yersin H. (2009). Gold(I) complexes bearing P∩N-ligands: An unprecedented twelve-membered ring structure stabilized by aurophilic interactions. Z. Naturforsch. B.

[98] Bondi A. (1964). van der waals volumes and radii. J. Phys. Chem..

[99] van Lenthe E., Baerends E.J., Snijders J.G. (1993). Relativistic regular two-component Hamiltonians. J. Chem. Phys..

[100] Wang F., Ziegler T., van Lenthe E., van Gisbergen S., Baerends E.J. (2005). The calculation of excitation energies based on the relativistic two-component zeroth-order regular approximation and time-dependent density-functional with full use of symmetry. J. Chem. Phys..

[101] ADF2014, SCM, Theoretical Chemistry, Vrije Universiteit, Amsterdam, The Netherlands. http://www.scm.com.

[102] van Lenthe E., Baerends E.J. (2003). Optimized Slater-type basis sets for the elements 1-118. J. Comput. Chem..

[103] Chen L.X., Shaw G.B., Novozhilova I., Liu T., Jennings G., Attenkofer K., Meyer G.J., Coppens P. (2003). MLCT state structure and dynamics of a Copper(I) diimine complex characterized by pump-probe X-ray and laser spectroscopies and DFT calculations. J. Am. Chem. Soc..

[104] Siddique Z.A., Yamamoto Y., Ohno T., Nozaki K. (2003). Structure-dependent photophysical properties of singlet and triplet metal-to-ligand charge transfer states in Copper(I) bis(diimine) compounds. Inorg. Chem..

[105] Iwamura M., Takeuchi S., Tahara T. (2007). Real-time observation of the photoinduced structural change of bis(2,9-dimethyl-1,10-phenanthroline) Copper(I) by femtosecond fluorescence spectroscopy: A realistic potential curve of the Jahn-Teller distortion. J. Am. Chem. Soc..

[106] Pye C.C., Ziegler T. (1999). An implementation of the conductor-like screening model of solvation within the Amsterdam density functional package. Theor. Chem. Acc..

[107] Mori K., Goumans T.P.M., van Lenthe E., Wang F. (2014). Predicting phosphorescent lifetimes and zero-field splitting of organometallic complexes with time-dependent density functional theory including spin-orbit coupling. Phys. Chem. Chem. Phys..

[108] Toptygin D. (2003). Effects of the solvent refractive index and its dispersion on the radiative decay rate and extinction coefficient of a fluorescent solute. J. Fluoresc..

[109] Niehaus T.A., Hofbeck T., Yersin H. (2015). Charge-transfer excited states in phosphorescent organo-transition metal compounds: A difficult case for time dependent density functional theory?. RSC Adv..

[110] Rössler U., Yersin H. (1982). Destabilization of a self-trapped exciton in a quasi-one-dimensional semiconductor: Mg[Pt(CN)_4_]_7_ H_2_O with hydrostatic pressure. Phys. Rev. B.

[111] Gliemann G., Yersin H. (1985). Spectroscopic properties of the quasi one-dimensional tetracyanoplatinate(II) compounds. Clusters.

[112] Tsuboyama A., Kuge K., Furugori M., Okada S., Hoshino M., Ueno K. (2007). Photophysical properties of highly luminescent Copper(I) halide complexes chelated with 1,2-bis(diphenylphosphino)benzene. Inorg. Chem..

[113] Turro N.J. (1978). Modern Molecular Photochemistry of Organic Molecules.

[114] Leitl M.J., Krylova V.A., Djurovich P.I., Thompson M.E., Yersin H. (2014). Phosphorescence versus thermally activated delayed fluorescence. Controlling singlet-triplet splitting in brightly emitting and sublimable Cu(I) compounds. J. Am. Chem. Soc..

[115] Murov S.L., Carmichael J., Hug G.L. (1993). Handbook of Photochemistry.

[116] Shafikov M.Z., Zaytsev A.V., Kozhevnikov V.N. (2021). Halide-enhanced spin-orbit coupling and the phosphorescence rate in Ir(III) complexes. Inorg. Chem..

[117] Yersin H., Strasser J. (2000). Triplets in metal–organic compounds. Chemical tunability of relaxation dynamics. Coord. Chem. Rev..

[118] Bergmann L., Hedley G.J., Baumann T., Bräse S., Samuel I.D.W. (2016). Direct observation of intersystem crossing in a thermally activated delayed fluorescence Copper complex in the solid state. Sci. Adv..

[119] Tschierlei S., Karnahl M., Rockstroh N., Junge H., Beller M., Lochbrunner S. (2014). Substitution-controlled excited state processes in heteroleptic Copper(I) photosensitizers used in hydrogen evolving systems. Chem. Phys. Chem..

[120] Garakyaraghi S., Danilov E.O., McCusker C.E., Castellano F.N. (2015). Transient absorption dynamics of sterically congested Cu(I) MLCT excited states. J. Phys. Chem. A.

[121] Iwamura M., Takeuchi S., Tahara T. (2015). Ultrafast excited-state dynamics of Copper(I) complexes. Acc. Chem. Res..

[122] Eng J., Penfold T.J. (2020). Understanding and designing thermally activated delayed fluorescence emitters: Beyond the energy gap approximation. Chem. Rec..

[123] Penfold T.J., Gindensperger E., Daniel C., Marian C.M. (2018). Spin-vibronic mechanism for intersystem crossing. Chem. Rev..

[124] Striplin D.R., Crosby G.A. (1994). Nature of the emitting ^3^MLCT manifold of rhenium(I)(diimine)(CO)_3_Cl complexes. Chem. Phys. Lett..

[125] Finkenzeller W.J., Thompson M.E., Yersin H. (2007). Phosphorescence dynamics and spin-lattice relaxation of the OLED emitter Ir(btp)2(acac). Chem. Phys. Lett..

[126] Finkenzeller W.J., Hofbeck T., Thompson M.E., Yersin H. (2007). Triplet state properties of the OLED emitter Ir(btp)_2_(acac): Characterization by site-selective spectroscopy and application of high magnetic fields. Inorg. Chem..

[127] Tinti D.S., El-Sayed M.A. (1971). New techniques in triplet state phosphorescence spectroscopy: Application to the emission of 2,3-dichloroquinoxaline. J. Chem. Phys..

[128] Sheldrick G.M. (1997). SHELXS-97, Program for the Solution of Crystal Structures.

[129] Sheldrick G.M. (1990). Phase annealing in SHELX-90: Direct methods for larger structures. Acta Crystallogr..

[130] Altomare A., Cascarano G., Giacovazzo C., Guagliardi A. (1993). Completion and refinement of crystal structures with SIR 92. J. Appl. Crystallogr..

[131] Sheldrick G.M. (1997). SHELXL-97, Program for Crystal Structure Refinement.

[132] Sheldrick G.M. (2008). A short history of SHELX. Acta Crystallogr..

[133] Sheldrick G.M. (2015). SHELXT–Integrated space-group and crystal-structure determination. Acta Crystallogr..

[134] Turbomole V6.4 2012. A Development of University of Karlsruhe and Forschungszentrum Karlsruhe GmbH, 1989-2007, TURBOMOLE GmbH, since 2007. http://www.turbomole.com.

[135] Weigend F., Ahlrichs R. (2005). Balanced basis sets of split valence, triple zeta valence and quadruple zeta valence quality for H to Rn: Design and assessment of accuracy. Phys. Chem. Chem. Phys..

[136] Stephens P.J., Devlin F.J., Chabalowski C.F., Frisch M.J. (1994). Ab initio calculation of vibrational absorption and circular dichroism spectra using density functional force fields. J. Phys. Chem..

[137] Peterson K.A., Figgen D., Goll E., Stoll H., Dolg M. (2003). Systematically convergent basis sets with relativistic pseudopotentials. II. Small-core pseudopotentials and correlation consistent basis sets for the post- d group 16–18 elements. J. Chem. Phys..

